# Travoprost Liquid Nanocrystals: An Innovative Armamentarium for Effective Glaucoma Therapy

**DOI:** 10.3390/pharmaceutics15030954

**Published:** 2023-03-15

**Authors:** Mohamed A. El-Gendy, Mai Mansour, Mona I. A. El-Assal, Rania A. H. Ishak, Nahed D. Mortada

**Affiliations:** 1 Department of Pharmaceutics and Pharmaceutical Technology, Faculty of Pharmacy, Future University in Egypt, Cairo 11835, Egypt; 2 Department of Pharmaceutics and Industrial Pharmacy, Faculty of Pharmacy, Ain Shams University, Cairo 11566, Egypt

**Keywords:** ocular delivery, liquid crystalline nanostructures, glaucoma, Travoprost, D-optimal design, ex vivo permeation, pharmacokinetics, pharmacodynamics

## Abstract

To date, the ophthalmic application of liquid crystalline nanostructures (LCNs) has not been thoroughly reconnoitered, yet they have been extensively used. LCNs are primarily made up of glyceryl monooleate (GMO) or phytantriol as a lipid, a stabilizing agent, and a penetration enhancer (PE). For optimization, the D-optimal design was exploited. A characterization using TEM and XRPD was conducted. Optimized LCNs were loaded with the anti-glaucoma drug Travoprost (TRAVO). Ex vivo permeation across the cornea, in vivo pharmacokinetics, and pharmacodynamic studies were performed along with ocular tolerability examinations. Optimized LCNs are constituted of GMO, Tween^®^ 80 as a stabilizer, and either oleic acid or Captex^®^ 8000 as PE at 25 mg each. TRAVO-LNCs, F-1-L and F-3-L, showed particle sizes of 216.20 ± 6.12 and 129.40 ± 11.73 nm, with EE% of 85.30 ± 4.29 and 82.54 ± 7.65%, respectively, revealing the highest drug permeation parameters. The bioavailability of both attained 106.1% and 322.82%, respectively, relative to the market product TRAVATAN^®^. They exhibited respective intraocular pressure reductions lasting for 48 and 72 h, compared to 36 h for TRAVATAN^®^. All LCNs exhibited no evidence of ocular injury in comparison to the control eye. The findings revealed the competence of TRAVO-tailored LCNs in glaucoma treatment and suggested the potential application of a novel platform in ocular delivery.

## 1. Introduction

Since their discovery, liquid crystalline nanostructures (LCNs) have gained esteem as nanoparticle systems for drug delivery. LCNs are typically composed of amphiphilic lipids such as monoolein (MO), additionally well-known as glycerol monooleate (GMO), and phytantriol (PYT), along with stabilizers such as Poloxamer 407. As a result, they make tremendous self-assembling entities for encapsulating both lipophilic and hydrophilic substances [1]. LCNs can be beneficial for ophthalmic drug delivery, where the high surface area of LCNs could endorse adhesion and hence drug penetration through the corneal epithelium, allowing for greater bioavailability. However, and to our best knowledge, LCNs are scarcely exploited for ocular administration [2,3,4,5,6,7,8,9,10,11,12,13,14,15]. Moreover, the integration of new stabilizers other than P407 is still limited in research.

Glaucoma is a set of progressive eye illnesses due to the rise of intraocular pressure (IOP), which ranges from 10 to 24 mmHg in a healthy human eye [16]. Elevated intraocular pressure (IOP) can lead to many eye complications. This elevation may produce plodding harm to the optic nerve, where the continuous long-term injury may result in a failure of communications between the retina and the brain and finally a loss of vision. Glaucoma is the second-leading cause of visual loss after cataracts. Different measures, such as laser treatment, surgery, and pharmacological treatment, can reduce IOP. Medicines such as adrenergic agonists, inhibitors of carbonic anhydrase, beta-blockers, analogs of prostaglandin, and hyperosmotic and myotic medicines are encompassed in the medicinal treatment of glaucoma. These medication types either increase the fluid flow from the eye or reduce the eye fluid generation. The foremost line of treatment for glaucoma in most countries is prostaglandin analogs; Latanoprost (Xalatan^®^), Travoprost (Travatan^®^), and Bimatoprost (Careprost^®^) are examples [17]. The prostaglandin analogs thereby reduce the IOP in the eye by increasing the draining of the aqueous humor.

Travoprost (TRAVO) differs from the other marketed prostaglandin analogs in being a full agonist at the prostaglandin F receptor (FP receptor), whereas the others are partial agonists with lower efficacy [18]. TRAVO is an isopropyl ester prodrug [17] hydrolyzed to the biologically active free acid by corneal esterases upon absorption into the eye after topical ocular administration. Despite its efficacy, Travoprost suffers from several major drawbacks as it is extremely hydrophobic with a log P of 4.6, has poor aqueous solubility of 7.59 × 10^−3^ g/L, and has a rapid terminal elimination half-life of approximately 45 min, thus affecting its ocular bioavailability [3,19].

In the literature, TRAVO has been loaded into a number of nanocarriers aiming to improve its corneal penetrability, ocular bioavailability, and hence its therapeutic efficacy. TRAVO-loaded nanoemulsion prepared by Ismail et al., 2020, showed an enhancement in the drug absorption better than that of the commercially available product Travatan^®^, as demonstrated by better bioavailability in terms of Cmax and AUC, and it also sustained the IOP lowering period. Moreover, the nanoemulsion formulation has been proven to be safe and nonirritant to ocular surfaces [3]. Self-assembled lipid DNA nanoparticles (NPs) were prepared by Schnichels et al. (2020) and loaded with TRAVO for glaucoma treatment. After eye drop instillation, TRAVO-NPs showed a prolonged adherence time to the eye that extended to one hour. Furthermore, the pharmacokinetic results disclosed that TRAVO-NPs delivered at least twice the drug quantity that was delivered by the free drug [20]. Additionally, Lambert et al. (2015) prepared a nano-sponge (NS) and examined the efficacy after intravitreal administration with the aim to lower the IOP in mice. The NS were loaded either with Brimonidine (an alpha-adrenergic agonist) or TRAVO, then their efficacy was compared. NS loaded with Brimonidine lowered IOP up to 30% for a duration of 6 days, whereas TRAVO-NS lowered IOP by about 19% to 29% for 4 days when compared to saline solution injection [21].

Yet, neither the ocular application of LCNs nor the impact of incorporating penetration enhancers (PEs) has been well reconnoitered. In addition, mounting the bioavailability of TRAVO with long-lasting effects was one of the challenges to be overcome. Hence, the present work focused on LCNs preparation and statistical optimization with the inclusion of new stabilizers and penetration enhancers. The optimized formulas were then chosen for the loading of the anti-glaucomic drug, Travoprost (TRAVO). Morphological examination, thermal behavior, and crystallinity studies were conducted. An ex vivo study was performed across the excised cornea of rabbits to assess the permeability of TRAVO-loaded LCNs. The selected medicated formulas were then exposed to in vivo pharmacodynamic and pharmacokinetic studies in comparison to Travatan^®^, which is the market product.

## 2. Materials and Methods

### 2.1. Materials

Glyceryl monooleate was generously provided by Danisco, Grindsted, Denmark. Poloxamer 407 (P407), Tween^®^ 80, Kolliphor^®^ HS 15 (formely regarded as Solutol^®^ HS), Cholesterol, Brij^®^ 52, Myrj^®^ S40, Acetonitrile, Phosphoric acid, Methanol (HPLC grade), Betamethasone, Dapoxetine, Tertiary butyl-methyl ether (TBME), Acetonitrile, Formic acid, Glacial acetic acid, Formalin, Ethyl alcohol, Xylol, Hematoxylin and Eosin (H & E) stain were purchased from Sigma Aldrich, St. Louis, Missouri, USA. Oleic acid was bought from Loba Chemie, Mumbai, India. Triglycerides of caprylic acid (Captex^®^ 8000) and mono/diglycerides of caprylic acid (Capmul^®^ MCM C8 EP/NF) were provided by the ABITEC Corporation, Columbus, OH, USA. El-Nasr Pharmaceuticals, Al Qalyubia, Egypt, supplied the sodium dihydrogen phosphate and sodium hydrogen phosphate. Lecithin^®^ (L-α-Phosphatidylcholine, egg about 72%) was procured from Fisher Scientific (Leicestershire, UK). Phytantriol (PYT) was kindly obtained from EVA Pharma, Giza, Egypt. Tocopherol polyethylene glycol 1000 succinate (TPGS) was kindly supplied by Isochem (Vert-Le-Petit, France). Travoprost (TRAVO) was kindly gifted from Orchidia Pharmaceutical Industries, Dakahlia, Egypt. Travatan^®^ eye drops (Novartis Pharmaceuticals UK Ltd., London, UK) were purchased from a community pharmacy. Fully-grown male New Zealand white rabbits, weighing 2.5 kg ± 0.5 kg, were granted from the Ophthalmology Research Institute, Giza, Egypt.

### 2.2. Preparation of Plain and TRAVO-Loaded LCNs

GMO, stabilizer, and penetration enhancer (PE) were melted in a beaker on a hot plate set to 60 °C to make plain LCNs. Then, using a heated plate with a magnetic stirrer (MS-300HS, Misung Scientific Co., Gyeonggi-do, Korea), the aqueous phase was poured into the lipid phase while being agitated at 500 rpm for 2 h. After that, a homogenizer was used to homogenize the dispersions for 1 min at 15,000 rpm (Silent Heidolph Crusher, Schwabach, Germany). The dispersion prepared was left at room temperature to be cooled down till it congealed, then kept at 5 ± 3 °C in a refrigerator, producing plain LCNs. For loaded LCNs, a calculated amount of TRAVO was weighed using an analytical balance (Sartorius CPA 225D, Gottingen, Germany) and added to the lipid phase, and the emulsification with the aqueous phase was then performed [22], producing TRAVO-LCNs with a concentration of 40 µg/mL equivalent to that of the marketed product Travatan^®^.

### 2.3. Experimental Design

Based on the results obtained from a preliminary study, the LCNs were prepared and optimized using a D-optimal experimental design, which allows a statistical evaluation of three independent variables, including the stabilizer amount (A) and the types of both PE (B) and stabilizer (C). GMO was the lipid of choice; in addition, three different stabilizer types were investigated: P407, Tween 80, and TPGS, all ranging in amounts from 1.25 to 25 mg, with or without the presence of a penetration enhancer (PE). Oleic acid, Captex^®^ 8000, and Capmul^®^ MCM were the PEs explored. Twenty formulations were prepared with different levels of variables as presented in Table 1. The dependent variables studied were the particle size (Y1), polydispersity index (Y2), and zeta potential (Y3) of the made LCNs.

### 2.4. Quantitative Analysis of TRAVO Using High-Performance Liquid Chromatography (HPLC)

TRAVO was quantitatively analyzed using HPLC (LC-20AT, Shimadzu, Japan) with the assay method implemented by the USP (USP 40, Travoprost Ophthalmic Solution Monograph). The mobile phase comprises a filtered and degassed mixture of deionized water, adjusted to pH 3 using phosphoric acid, and acetonitrile at a volume ratio of 35:65. The flow rate was adjusted to 1 mL/min, and the column temperature was set at 25℃. Under these chromatographic conditions, TRAVO was analyzed in the injected samples of 10 µL volume, detected at a UV wavelength of 220 nm, and eluted at a retention time of 3.5 min. According to ICH guidelines, the assay method’s linearity, accuracy, precision, limit of detection (LOD), and limit of quantitation (LOQ) were all verified.

### 2.5. Characterization of the Fabricated Travoprost Loaded LCNs

#### 2.5.1. Particle Size (PS), Polydispersity Index (PDI), and Zeta Potential (ZP)

The Malvern Zetasizer Nano Series (Malvern, Worcestershire, UK) was used to perform dynamic light scattering (DLS) on all prepared dispersions in order to obtain the average PS and PDI. The same device was used to measure the ZP values using the Laser Doppler Anemometry (LDA) technique. At 25 °C, measurements were carried out in triplicate. The samples’ counts ranged from 200 to 500 Kcps by dilution with deionized water [23].

#### 2.5.2. Determination of TRAVO Entrapment Efficiency (EE%)

Briefly, 500 µL of TRAVO-LCNs were loaded into the upper chamber of a Nano-sep^®^ (centrifuge tubes, Pall Life Sciences, Arizona, USA) and then centrifuged at 7000 rpm for 30 min using a cooling micro-centrifuge (Labogene-Scan speed 1524, GryozenCo., Ltd., Yuseoung-gu, Daejeon 305-301, Korea) adjusted at 4 °C. The concentration of free TRAVO in the supernatant collected in the lower chamber was determined quantitatively using the HPLC instrument, as previously described, using the following equation:

(1) $$EE\%=\frac{A-B}{A}\times 100$$

where A is the initial amount of the drug added and B is the amount that remained free in the supernatant.

#### 2.5.3. LCNs Morphology Investigation Using High-Performance Transmission Electron Microscopy (TEM)

F-1-L, F-3-L, F-4-L, and F-5-L optimum TRAVO-LCNs were chosen to investigate their morphological structures using HR-TEM. Imaging samples were obtained by adding 0.005 mL of the formula to a 300-mesh copper grid that was carbon-coated and placed on filter paper. The grid was given 3–5 min to dry at room temperature after the extra droplets were wiped away. Using an HR-TEM and a digital camera, the samples were photographed while remaining unstained and mounted on a holder.

#### 2.5.4. Crystallinity Study Using X-ray Powder Diffraction (XRPD)

For studying the crystallinity of the dried samples, the representative formulae were lyophilized by a freezer dryer (Christ, Alpha2-4LD Plus, Harz, Germany), which are plain GMO-based formulae (F-1-O, F-3-O, F-4-O, and F-5-O), with F-1-O composed of oleic acid as PE and Tween 80 as a stabilizer, while F-3-O, F-4-O, and F-5-O composed of Captex^®^ 8000 as PE and Tween 80, TPGS, and P407 as the stabilizer, respectively, were subjected to XRPD. The four formulae were compared to a dried PE-free formula composed of GMO and P407 as the lipid and stabilizer, respectively, which are the components of a typical cubic LCN. Cu-ka radiation, a voltage of 40 kV, and a current of 40 mA were used in an X-ray powder diffractometer (Philips, PW 3710, Caerphilly, United Kingdom). From 5° to 50° at 2Ɵ, all measures were implemented at a scan rate of 2°/min.

### 2.6. Ex Vivo Study for Corneal Permeation of TRAVO-Loaded LCNs

The ex vivo corneal penetration study was conducted on the eight optimized TRAVO-loaded LCN formulae (F-1-L to F-8-L) in comparison to drug solution (DS) using a customized Franz diffusion cell with an area of diffusion of 0.28 cm^2^ across excised rabbit corneas. The Research Ethics Committee of the Faculty of Pharmacy at Ain Shams University approved all animal procedures (acceptance number: REC-ASU 54).

Rabbits with average weights of 2.5 ± 0.5 kg were euthanized by urethane injection in the marginal ear vein. The oculomotor muscles, palpebral conjunctivas, and optic nerve plexus were trimmed by trained personnel using ophthalmic scissors, then the eyeballs were removed, and the corneas were excised after being cleaned. They were gently rinsed with simulated tear fluid (STF), composed of 6.7 g NaCl, 2.0 g NaHCO_3,_ and 0.08 g CaCl_2_ in 1 L of deionized water, pre-adjusted to a temperature of 34 °C. The fresh corneas were clamped between the two compartments of the Franz diffusion cell. A calculated volume of each formula, equivalent to 40 µg of TRAVO, was added to the donor compartment. Aliquots of 20 mL of STF were placed into the receptor compartment, and magnetic stirring at 50 rpm was allowed throughout the entire experiment. A sample of 300 µL was taken from each chamber at 0.5, 1, 2, 4, 6, and 8 h and replenished with fresh STF. The amount of drug permeated across the cornea was assayed by the validated method previously described using HPLC [7]. The cumulative amounts of TRAVO permeated per unit corneal area (µg/cm2) were measured and plotted against time.

Different corneal permeability parameters were measured from the permeation results, including steady-state flux (Jss) and permeability coefficient (Kp). The corneal permeation rate at steady state (Jss, µg/cm^2^/h) was determined from the slope of the linear part of the permeation curve. The apparent permeability coefficient (Kp) was calculated by the following equation:

Kp = Jss/Co

where Jss is the steady state flux and Co is the original concentration of the drug in the donor compartment [24].

### 2.7. Stability Study of the Selected TRAVO-Loaded LCNs

The physical stability of the selected TRAVO-LCNs (F-1-L and F-3-L) was evaluated by keeping them under refrigeration (5 ± 3 °C) for 90 days. The PS, PDI, ZP, and EE% were all evaluated before and after 30 and 90 days of storage, respectively.

### 2.8. Sterilization of TRAVO Loaded LCNs by Gamma Irradiation

The chosen TRAVO-LCNs (F-1-L and F-3-L) were exposed to gamma radiation using a ^60^Co radiation source at room temperature at a dosage rate of 5 kGy/h. The doses of radiation were 5, 10, 15, and 25 kGy. After being exposed to gamma rays, the physicochemical characteristics of the chosen formulae were evaluated, and their differences from non-irradiated samples were statistically compared [25].

The sterilization efficacy of the different gamma radiation doses applied to F-1-L and F-3-L was evaluated by a sterility test. The amounts of surviving bacteria and fungi were totaled to determine the suitable dose of gamma irradiation for sterilization. Sterility testing was carried out under aseptic conditions using fluid thioglycolate media, which is primarily intended for the culture of anaerobic bacteria but can also detect aerobic bacteria and soybean casein digest media, which are suitable for both fungi and aerobic bacteria. Each tested formula had two sterility test tubes, one as a negative control to check the sterility of the media used and the other as a positive control containing the tested formula itself. After a 14-day incubation period, the media was macroscopically examined for visual microbial growth and turbidity [25].

### 2.9. In Vivo Ocular Evaluation of the Selected TRAVO-Loaded LCNs

The optimized medicated formulae (F-1-L and F-3-L) were selected for in vivo evaluation as they showed the highest ocular flux and permeation.

#### 2.9.1. Pharmacodynamic Study in Rabbits Using Steroid-Induced Ocular Hypertension Model

The pharmacodynamic investigation included nine New Zealand white rabbits weighing approximately 2.5 kg ± 0.5 kg (3 rabbits per group) [26]. Throughout the test, the rabbits were maintained in isolated cages and supplied a conventional meal and water [26]. The rabbits were categorized randomly into three groups (G-I, G-II, and G-III). The left eyes served for induction and treatments, while the right ones served as negative controls (non-induced and non-treated). A corticosteroid injection was used to cause ocular hypertension (glaucoma). For 3 weeks, the rabbits in each group were given a 0.7 mL sub-conjunctival suspension of 0.1% betamethasone in the left eye [27]. When all rabbits’ intraocular pressure (IOP) was raised to the glaucomatous level (IOP > 24 mmHg) [3], 100 µL of either F-1-L or F-3-L was instilled once in the left eyes of G-I and G-II rabbits, respectively, and 100 µL of the marketed product Travatan^®^ eye drops was instilled once in the left eyes of G-III rabbits. A tonometer (Schiötz tonometer, Rudolf Riester GmbH, Germany) with a 10 g plunger load was used to measure IOP in the three groups. The plunger of the tonometer was pressed against the center of the cornea, and the reading was then taken once the disc was carefully lowered to the corneal surfaces [28]. The measurements were performed initially and at time intervals of 0.5, 1, 2, 4, 6, 8, 10, 12, 24, 36, 48, 60, and 72 h after the initial dose instillation, and the IOP values were recorded. Calculation of the percent of IOP reduction took place at each time interval (t) for each treatment group based on the initial IOP value (after induction) using the following equation:

(2) $$\%IOP\;Reduction=\frac{IOP\left(initial\right)-IOP(t)}{IOP(initial)}\times 100$$

#### 2.9.2. Pharmacokinetic Study in Rabbits

A tiny needle was placed across the cornea, right above the corneoscleral limbus in the anterior chamber of the eye, to extract drug-free aqueous humor from healthy rabbits. The samples were frozen in vials at −20 °C for later analysis and then thawed at room temperature for LC-MS/MS calibration curve construction. After administration of 100 µL of F-1-L, F-3-L, and Travatan^®^ to groups G-I, G-II, and G-III, respectively, samples of aqueous humor (50 µL) were withdrawn from the rabbits’ eyes at intervals of 0.5, 1, 2, 4, 6, 8, 12, 24, and 48 h. Samples were collected by a small needle inserted into the anterior chamber and stored at −20 °C for LC-MS/MS quantification of the drug. The pharmacokinetic parameters calculated were the maximum concentration of the drug in the aqueous humor (Cmax), the time needed to reach the maximum concentration (Tmax), the mean residence time (MRT), and the area under the concentration–time curve (AUC_0-48_ and AUC_inf_). The relative bioavailability (F%) was also calculated for each LCN formulation with respect to Travatan^®^ by the following equation:

(3) $$Relative\;Bioavailability(F\%)=\frac{AUC(inf)\;of\;LCN\;formula}{{AUC(inf)\;of\;Travatan}^{®}}$$

##### Quantitative Determination of TRAVO Using LC-MS/MS

Samples of the aqueous humor with a volume of 0.5 mL were placed in tubes made of glass with a volume of 7 mL. Internal standard (IS) solution (100 ng/mL Dapoxetine) was added at a volume of 50 µL. The vortex took place for 1 min for the samples. The rocker-mixer Reax II was used to mix the extraction solvent made of (4 mL tertiary butyl-methyl ether (TBME)) with the samples for a 10 min duration. Samples were centrifuged at 1790 *g* for 10 min at 4 °C, and the upper organic layer was moved into fresh tubes and evaporation took place using a vacuum concentrator until dry. A specific volume of mobile phase was added to the formed dry residues, then the reconstituted samples were mixed in a vortex for a minute and finally analyzed using LC-MS/MS (4500 LC-MS/MS MASS SPECTROMETER, AB SCIEX INSTRUMENTS, Concord, Ontario, L4K, 4V8, Canada). A sample volume of 10 µL was injected into an LC system using a C18 column. The isocratic mobile phase, composed of 80% acetonitrile and 20% water containing 0.1% formic acid, was delivered at a flow rate of 1.0 mL/min into the mass spectrometer’s electrospray ionization chamber. MS/MS detection in positive ion mode was used to analyze TRAVO and Dapoxetine (IS) by operating a mass spectrometer furnished with a Turbo Ion Spray Interface with a voltage set at 5500 V at 500 °C. The ions were identified using the multiple reactions monitoring (MRM) mode. Analyst software (version 1.4.2) was used to process the analytical data [29].

#### 2.9.3. Ocular Tolerability

The Draize test was implemented to compare the ocular safety of the chosen TRAVO-LCN formulations to the commercially available eye product (Travatan^®^). This was achieved by assessing the administered preparation’s potential irritating effects. After the solution was delivered into the rabbit’s eye, the potential for corneal, iridial, and conjunctival injury was evaluated. The glaucomatous eyes of rabbits treated with the tested formulations were examined for symptoms of redness, swelling, ulceration, or blindness using the three groups from the prior pharmacodynamic investigation [28,30]. The animals’ right eyes served as negative controls in all three groups. After the initial dose, rabbits were examined at predetermined time intervals of 1 h, 24 h, 72 h, and 14 days. Conjunctival redness or chemosis, iris inflammation, and corneal opacity were graded on a scale of 0–4, 0–2, 0–3, and 0–4. The lower the score, the less harmful the formulation [31].

#### 2.9.4. Histopathological Examinations

The safety of the specified TRAVO-LCN formulas on ocular tissues was confirmed through histopathological testing in comparison to the positive glaucomatous control eye. After euthanasia, tissue samples from the rabbits’ eyes were obtained. After that, they were fixed with Davidson’s Solution, which was made up of 300 mL 95% ethyl alcohol, 100 mL glacial acetic acid, 200 mL 10% neutral buffered formalin, and finally 300 mL distilled water [32]. After enucleation and trimming, the eyes were immediately placed in the solution. To keep the eye immersed, a gauze pad was employed. The globe was kept in the solution for 24 h before being removed and placed in 10% formalin. Trimmed tissue samples were cleaned and dehydrated in alcohol. After that, the dehydrated samples were cleaned in xylene, fixed in paraffin blocks, and sectioned at a thickness of 4–6 μm. For histological analysis, under a light optical microscope (Olympus Venox-S, AH-2, Tokyo, Japan), the acquired tissue sections were deparaffinized with xylol, and staining with H&E took place [33].

### 2.10. Statistical Analysis

All results were expressed as mean ± standard deviation (SD). All experimental data were statistically evaluated and optimized based on a D-optimal design using software named Design-Expert^®^ (Version 7, Stat-Ease Inc., Minneapolis, Minnesota, USA). The generated equation models were checked for validation by comparing the experimental results with the predicted ones. The following equation was used for the calculation of the prediction error:

(4) $$Prediction\;error=\frac{Predicted-Experimental}{Experimental}\times 100$$

Statistical analysis of the results was performed for all pharmacokinetic parameters by employing one-way analysis of variance (ANOVA) in SPSS software (IBM 20).

## 3. Results and Discussion

### 3.1. Experimental Design

Considering the preliminary study’s findings (Tables S1–S3, Supplementary Data), a D-optimal design was implemented, and twenty LCN formulas were prepared by varying the stabilizer amounts ranging from 1.25 to 25 mg (Factor A), in the absence or presence of various PEs (Factor B: None, oleic acid, Captex^®^ 8000, Capmul^®^ MCM), and different types of stabilizers (Factor C: P407, Tween 80, TPGS), aiming to select the optimized LCN formulations in terms of PS, PDI, and ZP for further drug loading. The average values of the measured responses (PS (Y1), PDI (Y2), and ZP (Y3)) for the prepared LCN formulations according to the experimental design are presented in Table 1.

The data revealed that nano-sized particles were formed with PS varying between 109.08 ± 6.53 and 666.35 ± 60.83 nm, where the lowest PS value was obtained by F8 containing the highest amount of TPGS (25 mg) as a stabilizer without any PE, while the highest one was obtained by F2 containing the lowest amount (1.25 mg) of Tween 80 and no PE as well. It can be noticed that PS of TPGS-stabilized formulae were rather comparable to those of P407-LCNs, where their respective sizes ranged between 109.08 ± 6.53 and 346.45 ± 30.05 nm, 147.78 ± 11.14, and 333.83 ± 51.39 nm, while those containing Tween exhibited higher PS ranging from 176.03 ± 5.91 to 666.35 ± 100.83 nm. In addition, it is obvious that the inclusion of a higher or lower amount of stabilizer revealed inconsistent results, which depended on the type of stabilizer and/or PE used.

The obtained PDI values varied from 0.15 ± 0.04 to 0.61 ± 0.07, indicating good PS distribution. It can be noticed that the respective PDI data of Tween, TPGS, and P407-stabilized LCN formulae ranged between 0.37 ± 016 and 0.58 ± 0.17, 0.33 ± 0.05 and 0.46 ± 0.01, and 0.15 ± 0.04 and 0.61 ± 0.07, noting the large variability in size distributions, particularly in the case of formulations prepared with P407.

Finally, the ZP results of all prepared LCN formulae showed high negative magnitudes ranging from −15.18 ± 0.95 to −81.03 ± 7.71 mV, indicating a high electrostatic stabilization. The obtained data revealed higher negativity values and hence better particle-particle repulsion and stability for both Tween 80 and TPGS-based formulations than those prepared using P407, as the respective ZP values ranged between −26.40 ± 1.27 and −72.70 ± 6.88 mV, −26.20 ± 2.51 and −81.03 ± 7.71 mV, and −15.18 ± 0.95 and −66.65 ± 5.72 mV.

#### 3.1.1. Data Analysis

##### PS Response

In influencing the effectiveness of cellular absorption and the biodistribution of the nanocarriers, PS is one of the most important parameters [34,35]. In general, small-sized monodispersed nanosystems are preferred over large ones, as the latter may result in ocular irritation and discomfort [36]. PS is an important feature for ocular delivery as it affects intraocular penetration. Membrane permeability decreased with the increase in PS, where particles < 400 nm showed greater penetration through the corneal mucosa [8].

**Figure 1:**
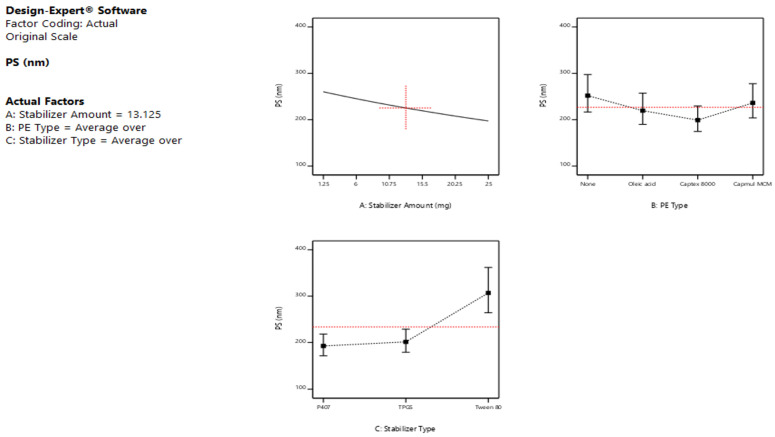
Main effect plots showing the effect of the independent variables; (**A**) stabilizer amount, (**B**) penetration enhancer type, and (**C**) stabilizer type on the particle size of the prepared LCNs.

The ANOVA results (Table 2) of the PS response showed that both the stabilizer amount (A) and type (C) significantly affected the sizes of the formed LCNs (*p* < 0.05), while the type of PE (B) revealed a non-significant effect on PS (*p* > 0.05). The main effect plots illustrated in Figure 1 showed that upon increasing the amount of stabilizer from 1.25 to 25 mg, the average PS of the produced LCNs decreased significantly (*p* < 0.05), irrespective of the type of stabilizer. This is ascribed to the fact that the stabilizer acts as a size-controlling agent, which prevents the growth and coalescence of nanoparticles. The obtained result is in agreement with the findings of Das et al. [37], who formulated silver nanoparticles using different stabilizer concentrations. The results revealed that the particles prepared without using any stabilizer are coarser in size than those prepared using a stabilizer. Ishak et al., 2017, also found that the higher amount of Tween 80 led to a significant decrease in the PS of the prepared nanocarriers [38]. Mansour et al., 2017 [39] also noticed that the PS was significantly increased (*p* < 0.05) by increasing the amount of lipid while keeping the amount of P407 constant, or, in other words, the PS decreased as the lipid: P407 weight ratio decreased, as described by Nakano et al., 2001 [40]. This may be explained by the fact that the stabilizers utilized are hydrophilic polymers or surfactants, which improve the positive curvature of cubosomes while decreasing their negative curvature when compared to hydrophobic substances such as lipids [39]. According to the nucleation and growth model described by Lamer and Dinegar, a low stabilizer concentration might reduce the nucleation and lower the formation of an enormous number of nuclei and henceforward aid the growth of larger nanoparticles [41].

Further inspection of the main effect plot Figure 1 revealed that the stabilizer type exhibited a substantial impact on the average PS of the prepared LCNs (*p* < 0.05). Changing the stabilizer type was associated with variable effects, as the addition of Tween 80 caused a buildup in LCN size when compared to P407 and TPGS, which showed comparable smaller PS under the same formulation conditions. These results are consistent with Dibaei et al. (2019) [42], who revealed that using TPGS as a stabilizer in curcumin-loaded nanosuspensions produced NPs exhibiting the lowest PS among all the prepared NPs. Since P407 has a longer hydrophobic alky chain length than Tween 80, they showed smaller PS as the emulsifying effect was positively correlated to the emulsifier’s alkyl carbon chain length. In other words, the longer the alkyl carbon chain, the better the emulsifying effect [43].

In addition, the plots of Figure 1 demonstrated that the absence/presence of PE, as well as the type of PE, used did not have a significant impact on the mean sizes of the produced LCNs confirming the results of the ANOVA test.

All the two-way interactions, AC, BC, and AB exhibited no significant influences on the PS of LCNs with *p* values >0.05.

##### PDI Response

Based on the ANOVA results (Table 2), it was obvious that the PDI was significantly affected only by the stabilizer type (C) (*p* < 0.05), with no significant effect of both A and B factors (*p* > 0.05), i.e., the stabilizer amount and the type of PE. The 2-FI interactions AC and BC revealed significant effects on the size distributions of the formed LCNs (*p* < 0.05), while AB was not significant as the *p*-value exceeded 0.05.

**Figure 2:**
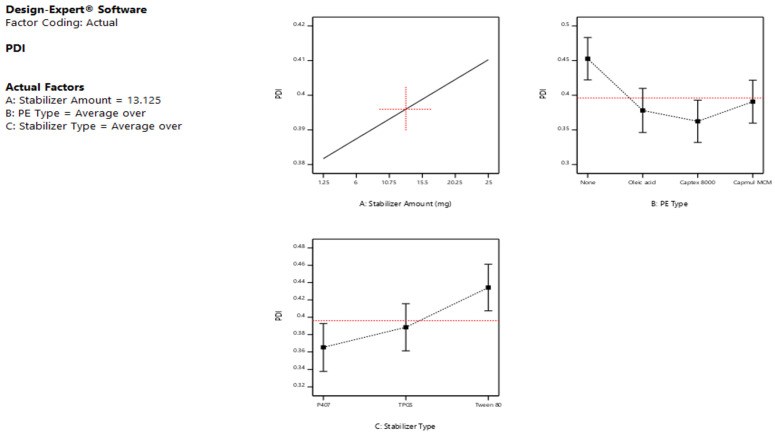
Main effect plots showing the effect of the independent variables; (**A**) stabilizer amount, (**B**) penetration enhancer type, and (**C**) stabilizer type on the PDI of the prepared LCNs.

Low PDI values indicate monodispersed nanoparticles, while higher ones [32] indicate polydispersed ones [39]. As obvious from the main effect plots presented in Figure 2, Tween 80-stabilized LCNs showed higher PDI values than those prepared with P407 and TPGS, confirming the significant effect of the stabilizer type on PDI. The increase in particle heterogeneity upon using Tween 80 could be attributed to the agglomerates or micelles composed of free stabilizers that did not share in the formation of cubosomes and hence decreased the homogeneity of the nano-dispersion [44]. Low PDI values were obtained using P407, indicating a uniform size and a good distribution of particles, which is in agreement with those obtained by Patil et al., 2019, who used P407 as a stabilizer while preparing cubosomes with GMO as a lipid [45].

##### Zeta Potential Response

The surface charge of LCNs is a crucial parameter for ensuring the stability of the generated nano-dispersions. The need for a reasonably stable formulation is highlighted by the reported fabrication of a physically stable LCN with a ZP value of at least −30 mV or −20 mV to be electrostatically stabilized or sterically stabilized systems, respectively [42,46]. The ANOVA results (Table 2) revealed that all three ZP model terms, A, B, and C, were significantly affecting the ZP values with *p* values < 0.05.

**Figure 3:**
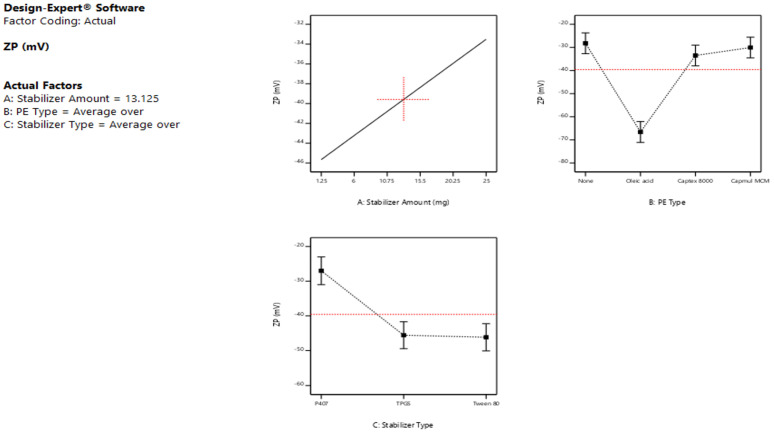
Main effect plots showing the effect of the independent variables; (**A**) stabilizer amount, (**B**) PE type, and (**C**) stabilizer type on the ZP of the prepared LCNs.

By observing Figure 3, a positive correlation occurs between the amount of the stabilizer and the corresponding ZP values, which is to say that increasing stabilizer content from 1.25 to 25 mg caused an increase in ZP values, which is to say a decrease in the ZP negative magnitudes. Our data are in agreement with earlier studies by Sun et al., 2004, who coated model nanoparticles with Tween 80 as a tool for delivering drugs to the brain. This may be because the adsorbed surface layer of non-ionic surfactant is probably masking the surface charge of the LCNs, as the more the adsorbed non-ionic surfactant is, the thicker the adsorbed layer and the more positive shift in ZP values [47].

Regarding the type of stabilizer, Tween 80 and TPGS showed higher negative magnitudes of ZP values compared to P407; this could refer to the capability of the latter to cover efficiently the surface of the formed particles owing to its high molecular weight (12,600 g/mol) compared to those of TPGS (1542 g/mol) and Tween 80 (1310 g/mol) [34]. The decline in ZP magnitudes suggests the formation of a stabilized polymer layer [48].

As for the PE type, the negativity of ZP values of oleic acid-based LCNs was significantly higher than both Captex^®^ 8000 and Capmul^®^ MCM-LCNs. This increase is brought on by the oleic acid molecules’ free carboxylic groups, which have negative charges [39]. The amphiphilic nature of these glycerides, which tend to be adsorbed onto the LCN surfaces, displaying a shielding effect, was also linked to the decreased ZP values of Captex 8000 and Capmul MCM [49].

#### 3.1.2. Model Validation and Optimization

For model validation and optimization, the optimized formulations were chosen according to the numerical optimization generated from the Design Expert^®^ software. This optimization process was conducted to optimize the LCN formulations based on formulation constraints: factor A: at the lowest and highest stabilizer amounts; factor B: at each PE type; and factor C: the stabilizer type in range. The target goals were adjusted as follows: (1) minimize PS, (2) minimize PDI, and (3) ZP <−25 mV. Eight formulae were then selected based on the highest desirability function (D) approaching unity. The optimized formulation compositions are presented in Table 3. The collected experimental findings were contrasted with those predicted, and the prediction error (% bias) for each response model was then determined. The experimental and predicted data and the calculated prediction errors are collected in Table 3. As shown, the results of the prediction error are all below 20%, confirming the validity and prediction capability of the three response models [50].

### 3.2. Preparation of TRAVO-Loaded LCNs

Based on the optimization results obtained from the D-optimal design, the eight optimized formulae were selected for drug loading. These formulations were loaded with TRAVO at a concentration of 40 µg/mL mimicking that of the marketed product Travatan^®^, and then coded F-1-L, F-2-L, F-3-L, F-4-L, F-5-L, F-6-L, F-7-L, and F-8-L. They were prepared by the addition of calculated drug amount to the melted lipid using the hot melt emulsification technique previously described. The prepared TAVO-loaded LCN formulations were then subjected to in vitro and ex vivo characterization.

### 3.3. Characterization of TRAVO-Loaded LCNs

#### 3.3.1. PS, PDI, and ZP

It can be noticed from Table 4 that all the loaded LCN formulae (F-1-L–F-8-L) showed a nano-sized range from 129.40 ± 11.73 to 361.57 ± 29.21 nm. The PDI values were within the acceptable range (< 0.5), indicating the size uniformity of the prepared formulations. Both formulations (F-1-L and F-3-L) containing Tween 80 as a stabilizer, although used at its highest amount (25 mg), showed variable PS; 216.20 ± 6.12 and 129.40 ± 11.73 nm, respectively. This is mostly due to the discrepancy in the PE used as oleic acid and Captex^®^ 8000, respectively. This may be attributed to the effect of Captex^®^ 8000, which acts as a surface-active agent due to its amphiphilic property, as it supports lowering the tension at the particle-water interface and hence reducing the sizes of the formed particles. P407-stabilized LCNs recorded small PS values for the formulae F-2-L, F-5-L, and F-6-L, although the lower stabilizer amounts were included. This warrants the effectiveness of P407 in LCN stabilization. In contrast, the formulations stabilized with TPGS (F-4-L, F-7-L, and F-8-L) revealed higher size results irrespective of the PE type used. The highest PS obtained in the case of F-7-L could be attributed to the lower amount of TPGS included (1.25 mg). All the optimized formulae loaded with TRAVO exhibited a wide range of ZP values ranging from −13.1 ± 1.27 to −72.9 ± 1.97 mV, noting that the higher negative magnitudes were recorded specifically for oleic acid-based LCNs as discussed before. The overall results guaranteed the high stability of the medicated LCN formulae.

#### 3.3.2. Entrapment Efficiency %

As noticed, all formulae possessed a high EE% of TRAVO, ranging from 71.29 ± 8.87 to 85.30 ± 4.29%. These results proved the high ability of LCNs to entrap hydrophobic drugs such as TRAVO even in the presence of different stabilizers and PEs. This could be attributed to the composition of the prepared LCNs, which include a high proportion of lipids, providing good compatibility with the hydrophobic drug (log P = 4.6). This is in agreement with the outcome revealed by Mansour et al., 2017 [39], who declared that cubosomes are capable of entrapping high loads of hydrophobic cargo.

#### 3.3.3. LCNs Morphology Examination Using TEM

**Figure 4:**
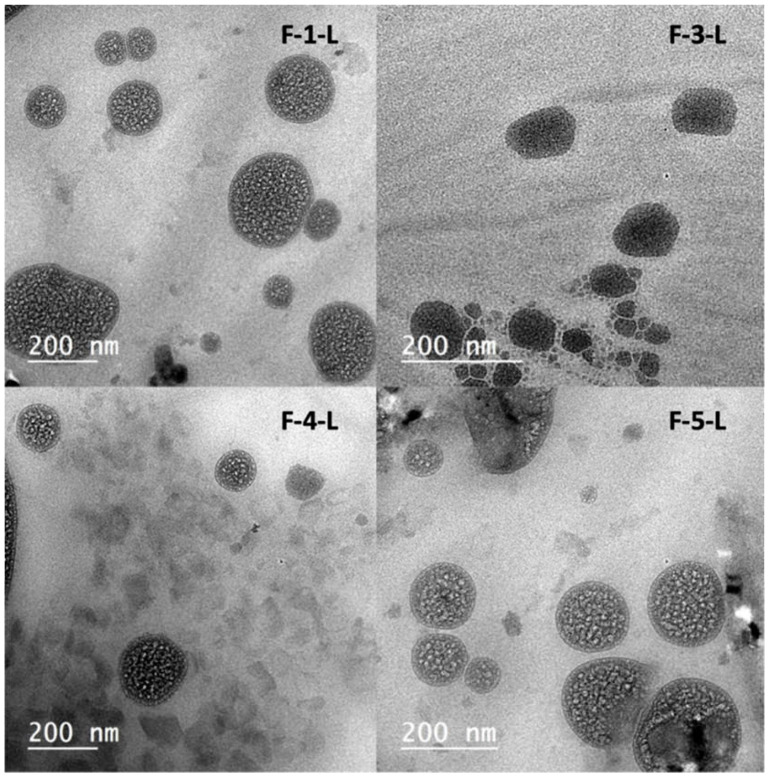
TEM photomicrographs showing the morphology of representative TRAVO-loaded LCNs (F-1-L, F-3-L, F-4-L, and F-5-L).

TEM was employed to examine the morphology of representative TRAVO-loaded LCN formulations (F-1-L, F-3-L, F-4-L, and F-5-L). As illustrated in Figure 4, all formulae exhibited irregular hexagonal to spherical structures. Because of the negative correlation between HLB and CPP, this could be explained by the effect of fatty substances with low HLB values, such as oleic acid, on increasing the curvature of the bicontinuous layer within the liquid crystal. Because of the negative correlation between HLB and CPP, this could be illuminated by the impact of fatty substances with low HLB, such as oleic acid, on increasing the curvature of the bicontinuous layer inside the liquid crystal [51]. Captex molecules with hydrophobic long chains of fatty acids may cause bigger hydrophobic volumes (Vs) in GMO-based cubosomes, resulting in a higher CPP value for the Captex^®^/GMO combination and encouraging a transition from the inverse cubic phase to the hexagonal phase [52]. This result matched the view stated by Mansour et al. in 2017 [39].

#### 3.3.4. Crystallinity Study Using X-ray Powder Diffraction (XRPD)

**Figure 5:**
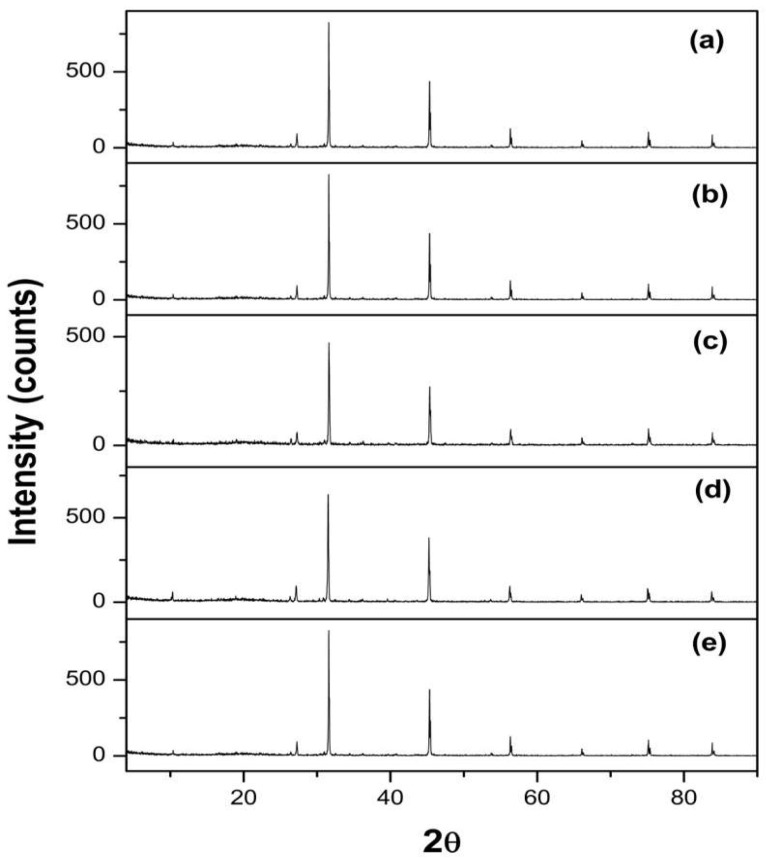
XRPD patterns of (**a**) reference formula, (**b**) F-1-O, (**c**) F-3-O, (**d**) F-4-O, and (**e**) F-5-O.

To confirm the crystallinity of different LCNs, XRPD is a useful method. X-ray diffractograms of the representative lyophilized unloaded LCNs (F-1-O, F-3-O, F-4-O, and F-5-O) are presented in Figure 5, compared to the reference formula prepared with GMO and P407 as the lipid and stabilizer, respectively. The X-ray diffractograms of different LCNs showed mutual peaks located at around 32°, 45°, 57°, 76°, and 84° 2θ, as shown in Figure 5. This may be distinctive of the liquid crystals produced, confirming the resemblance of their crystalline structures, as stated by Bei et al., who revealed almost comparable peaks in X-ray patterns [53].

### 3.4. Ex Vivo Study for Corneal Permeation of TRAVO-Loaded LCNs

**Figure 6:**
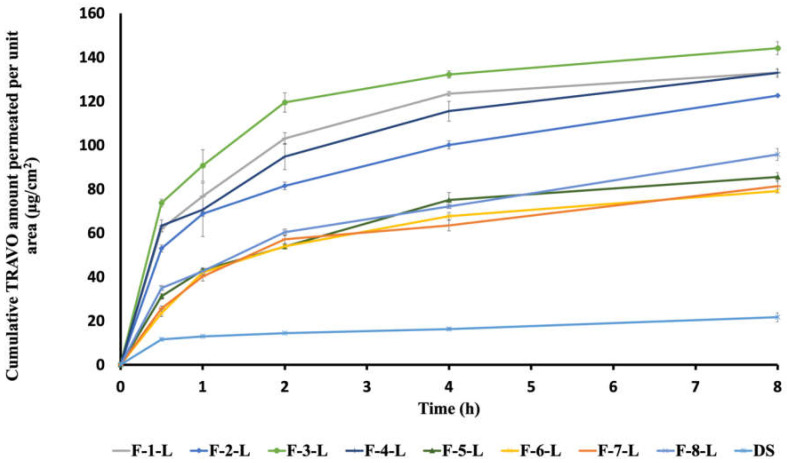
Ex vivo permeation profiles of TRAVO from different loaded LCNs in simulated tear fluid through excised rabbit cornea (mean ± SD, *n* = 3).

As obvious from Figure 6, the TRAVO-LCN formulae encoded F-1-L, F-3-L, and F-4-L showed the highest cumulative drug amount permeated after 8 h (Q_8_), reaching 132.94 ± 4.940, 144.07 ± 1.60 and 132.84 ± 4.55 µg/cm^2^, respectively. F-3-L showed a higher significant Q_8_ compared to that obtained by both formulas, F-1-L and F-4-L (*p* < 0.05). This was then followed with the LCN formula, F-2-L, which also exhibited a relatively high Q_8_ value of 122.54 ± 2.45 µg/cm^2^. However, the formulae, coded F-5-L, F-6-L, F-7-L, and F-8-L, revealed slower permeation profiles, as the respective Q_8_ data attained 85.40 ± 7.28, 79.00 ± 3.03, 81.28 ± 8.56, and 95.70 ± 9.47 µg/cm^2^, respectively. Drug solution (DS) showed the lowest Q_8_, reaching only 21.59 ± 7.17 µg/cm^2^, with a significant difference compared to the other formulas (*p* < 0.05).

One potential reason that LCNs might enhance corneal permeation is the bio-adhesive property of the liquid crystalline nanoparticles. Their small PS and increased surface area may also promote drug permeation across biological membranes. The nano-sized range and increased surface area of the prepared TRAVO-LCNs could promote adhesion and hence drug penetration through the corneal epithelium, allowing for greater drug delivery to the anterior eye. Furthermore, the structural similarities between the bicontinuous lipid bilayer architectures of cubosomal nanoparticles and corneal epithelial membranes allow membrane fusion and direct transit of the medication into the corneal cells, which may explain the improved penetration of LCN formulations [54]. Furthermore, the cubosomes’ main component, GMO, has strong penetration-enhancing properties via ocular membranes [15,55].

Furthermore, the permeation parameters, Jss and Kp, were determined based on the constructed ex vivo permeation profiles, and the results are presented in Table 5. The steady-state flux (Jss) was calculated from the slope of the linear portion of the permeation plots, and the permeation coefficients (Kp) were determined accordingly. As expected, both Tween 80-stabilized formulae, F-1-L and F-3-L, showed the highest fluxes and Kp values, while the LCN formulations stabilized with P407, encoded F-5-L and F-6-L, recorded the least values of permeation parameters, as shown in Table 5. Based on the obtained results, we can deduce the suitability of the optimized TRAVO-LCNs stabilized with Tween 80 coded F-1-L and F-3-L for enhanced ocular delivery. Therefore, these formulations were chosen for further studies.

### 3.5. Physical Stability of the Selected TRAVO-Loaded LCNs

The optimized medicated LCN formulae, F-1-L, composed of GMO, oleic acid, and Tween 80 as the lipid, PE, and stabilizer, respectively, and F-3-L, consisting of GMO, Captex^®^ 8000, and Tween 80 as the lipid, PE, and stabilizer, respectively, were stored for 90 days under refrigeration at 5 ± 3 °C. The PS, PDI, ZP, and EE% of the stored samples were determined and compared to the freshly prepared formulations. After storage, the stored LCNs were found to retain their parameters with non-significant variations compared to the fresh preparations (*p* > 0.05), as shown in Table S4 (Supplementary File). The resulting stability might be due to the use of Tween 80, which acts as a stabilizing agent during nanoparticle formation and reduces the surface energy, leading to the inhibition of crystal growth [56].

### 3.6. Sterilization of TRAVO-Loaded LCNs by Gamma Irradiation

The two potential LCN formulae, F-1-L and F-3-L, were sterilized using gamma irradiation and then tested for their sterility against the presence of any microbial contamination, either bacterial or fungal. Different radiation doses of 5, 10, 15, and 25 kGy were applied to sterilize the selected loaded formulas. After performing the sterilization process, PS, PDI, ZP, and EE% were re-tested, and the data are collected in Table S5 (Supplementary File). All formulae showed non-significant changes in their physical characteristics at all radiation doses (*p* > 0.05). To ensure the sterility of the chosen formulae and to establish the minimal dose (kGy) necessary to achieve their sterility, a confirmatory sterility test was carried out. The absence of any microbial growth in any of the examined samples verifies the formula’s sterility and the efficacy of gamma irradiation for sterilization, even at lower doses. Our findings were consistent with those of Youshia et al. (2021) [57].

### 3.7. In Vivo Ocular Evaluation of the Selected TRAVO-Loaded LCNs

In vivo studies were executed for the estimation of LCNs’ ability to deliver TRAVO effectively for glaucoma treatment. Pharmacodynamic and pharmacokinetic studies were performed in rabbits to evaluate the efficacy of the formulae; in addition, the safety of LCN formulae was assessed using the Draize test and histopathological examinations.

#### 3.7.1. Pharmacodynamic Study in Rabbits Using Steroid-Induced Ocular Hypertension Model

**Figure 7:**
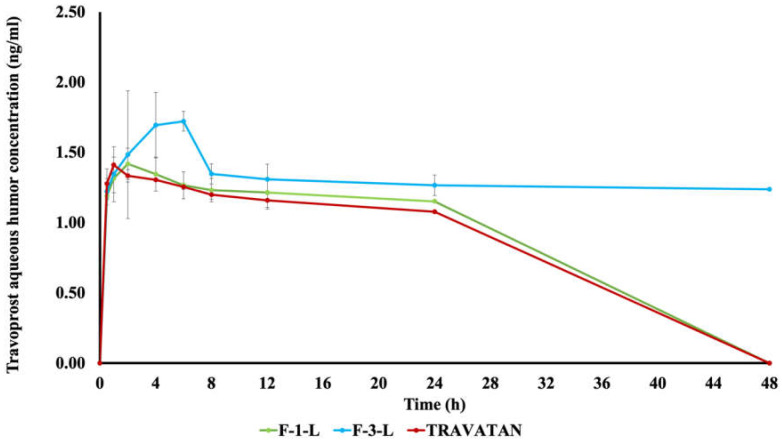
Aqueous humor TRAVO concentrations versus time post-application of F-1-L, F-3-L, and Travatan^®^ in the rabbits’ left eyes (mean ± S.D, *n* = 3).

The effectiveness of the selected medicated formulae, F-1-L and F-3-L, in lowering the elevated IOP and hence improving glaucoma was assessed in rabbits and then compared to the market product Travatan^®^ eye drops. As shown in Figure 7, the IOP measurements of the right rabbit eyes (non-induced and non-treated) served as negative controls and showed a normal range from 16.43 ± 1.75 to 20.22 ± 1.42 mmHg. After the induction of glaucoma, the initial IOP was measured and recorded at 37.20 mmHg, indicating the glaucomatous eye condition in the left rabbit eyes of all designated groups. After treatment, the eyes of G-I treated with TRAVO-LCNs ‘F-1-L’ attained their lowest IOP value (15.6 mmHg) at 6 h post-dose application and were shown to maintain the lowering effect for 48 h, after which the IOP starts to rise. However, the medicated formulation F-3-L revealed a much lower mean IOP measurement (13.9 mmHg) after 24 h post-treatment, which lasted during the whole experiment duration (72 h). On the other hand, the marketed product Travatan^®^ demonstrated a gradual reduction in IOP measurements, reaching its peak (14.7 mmHg) at 8 h and persisting for 36 h, after which the IOP significantly increases. As was obvious, the obtained results demonstrated the superiority of the optimized LNC formulations over the commercial product TRAVATAN in lowering IOP for a lasting duration. These results are in alignment with the ex vivo permeation results obtained. The sustained efficacy of the tested LCN formulations could be attributable to the controlled diffusion rate of the drug through the water channels within the nanocrystals [9]. Furthermore, the structural similarity between the bicontinuous lipid bilayer of LCNs and the corneal epithelial cells allows membrane fusion, permitting direct transit of the medication through the corneal cells, which elucidates the improved penetration of LCN formulations [54]. It is important to note that although Travatan^®^ eye drops contain propylene glycol, which acts as a cosolvent with permeation enhancer properties, it was shown to be insufficient to maintain the therapeutic effect of TRAVO for a longer period.

The percentages of IOP reduction were further calculated for each group at different time intervals based on initial IOP values; the data are presented in Table 6. Both LCN formulations, F-1-L and F-3-L, showed higher IOP reduction for a longer time than the commercial product; this could be due to the synergistic effect of GMO, Tween 80, and oleic acid/Captex^®^ as all LCN components are reported to manifest satisfactory penetration properties [58,59,60]. The discrepancy in the results of both LCN formulations could emphasize the importance of electrostatic interactions that might occur between the LCN particles and the cornea. As the cornea exhibits negative surface charges, it is reported that the particles with positive charges or less negative charges could be retained for a longer time, allowing for more drug penetration through the cornea [8]. Based on the ZP results obtained previously, F-3-L revealed a significantly lower mean ZP value of −17.55 ± 2.10 mV compared to −72.93 ± 1.97 mV recorded for F-1-L (*p* < 0.05), which in turn can assume a longer residence of the former formula onto the cornea surface, hence an enhanced therapeutic effect. Moreover, the size of the lipid particles is reported to have an influence on corneal permeation, affecting the pharmacological effect of the loaded drug [8]. The sizes of both tested LCN formulae were shown to be significantly different (*p* < 0.05) according to the data stated previously, as F-3-L demonstrated a much lower PS than F-1-L, where the respective sizes were 129.40 ± 11.73 and 216.20 ± 6.12 nm, warranting the enhanced permeation of F-3-L and hence its superiority in lowering IOP.

#### 3.7.2. Pharmacokinetic Study

The results of drug concentration in aqueous humor *versus* time are illustrated in Figure 7, and the calculated pharmacokinetic parameters are collected in Table 7. The LCN formula, F-3-L, showed a significantly higher C_max_ of 1.80 ± 0.15 ng/mL (*p* < 0.05) when compared to 1.46 ± 0.06 and 1.42 ± 0.09 ng/mL obtained in the cases of F-1-L and Travatan^®^, respectively. It is noted that the difference between the C_max_ of both F-1-L and Travatan^®^ was statistically non-significant (*p* > 0.05). The highest median T_max_ of 6 h attained by F-3-L confirmed more controlled drug permeation behavior than that achieved by Travatan^®^ and F-1-L, recording 1 and 2 h, respectively. Furthermore, the chosen formulation F-3-L showed significantly higher AUC_0-48_, AUC_inf,_ and MRT in comparison to the respective data obtained from F-1-L and Travatan^®^, as shown in Table 7. The formula F-1-L showed a relative bioavailability of 106.1%, while F-3-L showed a much higher value of 322.82% with respect to the market product. The obtained results are in good agreement with what was achieved in the pharmacodynamic study, confirming the sustained effect of F-3-L in lowering the IOP compared to F-1-L and Travatan^®^. The increased ocular bioavailability of LCNs may be due to three factors: the prolonged contact time of LCNs with ocular tissues, the drug’s ability to permeate across the cornea, and high drug loading capacity. Liu et al. have investigated pre-ocular retention and revealed that the LCN formulations caused a longer residency on the corneal membrane, potentially lengthening the ocular contact period. This in turn demonstrated a higher flux and permeability of the LCN formulations compared to the drug solution [9]. The unique structural features of LCNs, such as their high packing density and extraordinarily long linear water-filled channels that may hold more medication, may result in increased drug permeability through the cornea [9].

#### 3.7.3. Ocular Tolerability

**Figure 8:**
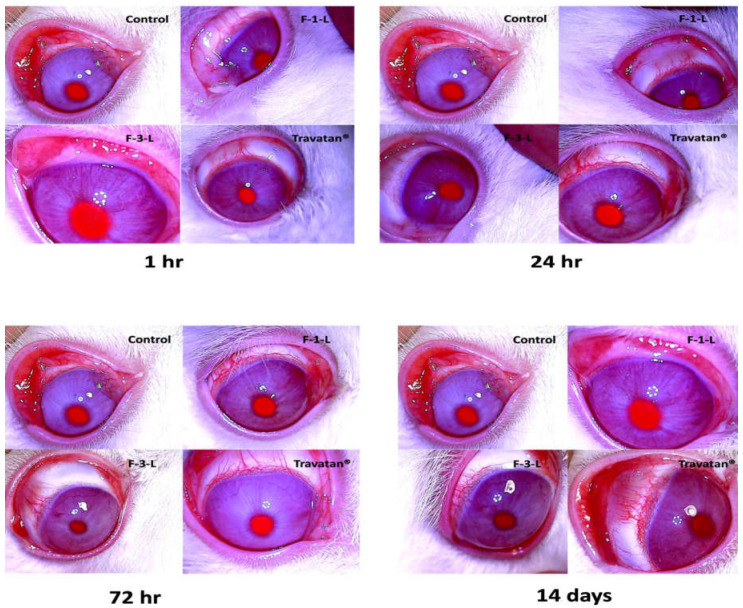
Images of rabbits’ eyes post-application of the tested samples. No physiological difference is observed between the eyes treated with the optimized LCNs (F-1-L) and (F-3-L) and the marketed product (Travatan^®^) compared to the control eye.

Ocular tolerability was performed using the Draize test, which is used for evaluating the toxicity of suspected eye irritants [61]. Ocular irritation (status of the cornea, iris, and conjunctiva) on the treated left eyes, was assessed at 1 h, 24 h, 72 h, and 14 days. As shown in Figure 8, none of the examined LCNs or the marketed product exhibit any symptoms of ocular injury.

#### 3.7.4. Histopathology Examinations

**Figure 9:**
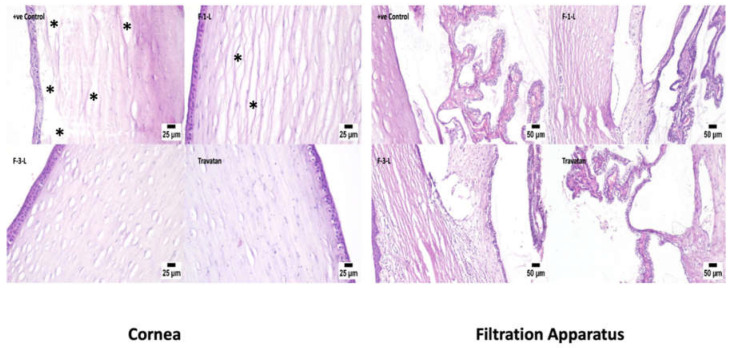

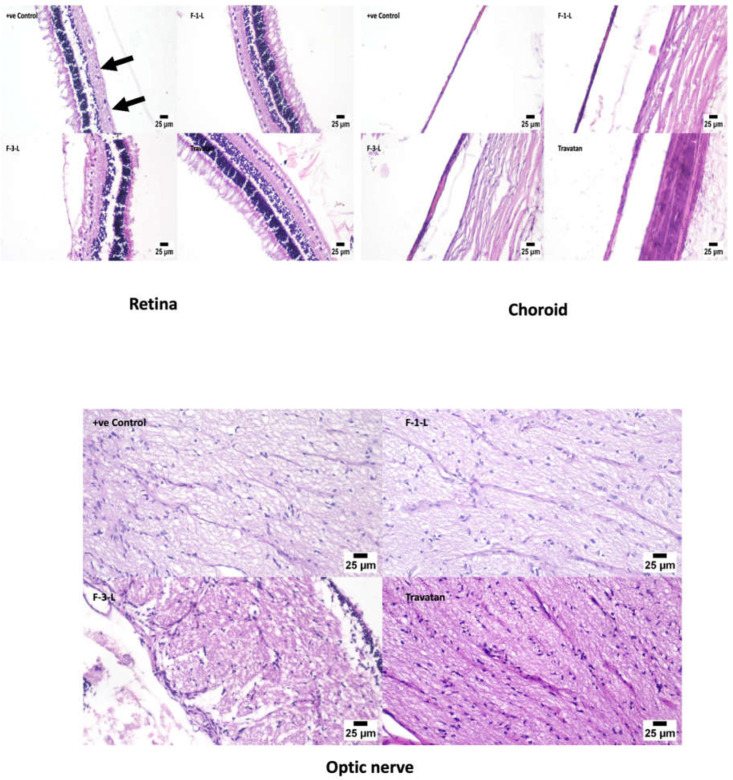
Photomicrographs of the eye of different sections of the tested samples. (*) denotes corneal edema, the black arrows point out undersized hyperchromatic ganglion cells with pyknotic nuclei.

Microscopic examinations of the eye were performed via assessment of the cornea, filtration angle, choroid, retina, and optic nerve. As shown in Figure 9, regarding the cornea, the positive glaucomatous control group showed marked corneal edema manifested by dispersion of the corneal stroma with edematous fluid. Meanwhile, the cornea receiving F-1-L showed mild corneal edema, while F-3-L showed marked improvement, and the cornea appeared apparently normal while the cornea treated with Travatan^®^ was histologically normal. As shown in Figure 9, histopathological changes in the filtration angle were seen in the positive control group, including thickening of the basement membrane with increased collagen deposition and ciliary muscle hyalinosis. Reduced cellular components, increased matrix and fibrillar components, and hyalinization of the trabecular meshwork were found in the meshwork. All other experimental groups revealed apparently normal filtration angles, ciliary bodies, and trabecular meshwork. Figure 9 is concerned with the vascular layer “choroid” of the eyes. The positive control group showed a compressed choroid. Regarding F-1-L, mild choroid compression was observed, while F-3-L and Travatan^®^ exhibited an apparently normal choroid. As shown in Figure 9, a generalized retinal atrophy with loss of inner ganglion cells was seen in the positive control group. The ganglion cells that remained were undersized and hyperchromatic, with pyknotic nuclei. The other experimental groups showed apparently normal retinas. As shown in Figure 9, the optic nerve of the positive control group showed vacuolation, while the optic nerve of the other groups exhibited the absence of histopathological alterations.

The results revealed that F-3-L, composed of GMO as a lipid, Tween 80 as a stabilizer, and Captex^®^ 8000 as PE, each weighing 25 mg and loaded with TRAVO at a concentration of 40 µg/mL, is the formula of choice as it showed the optimum results in pharmacodynamics and pharmacokinetics studies, accompanied by a high safety profile. The overall results indicate the supreme ability of LCNs to deliver TRAVO by the ocular route and improve glaucoma.

## 4. Conclusions

The present work describes the successful integration of an innovative ocular penetration enhancer (Captex 8000) into classical liquid crystalline nanostructures. Furthermore, Travoprost loading in these nanostructures resulted in a safe and effective approach to glaucoma treatment. The medicated liquid crystalline nanostructures illustrated favorable drug penetration power throughout the corneal layer, as well as efficient stability and high Travoprost entrapment efficiency. When compared to the amount delivered using the market product, Travatan^®^, the bioavailability of Travoprost was heightened threefold when delivered from liquid crystalline nanostructures. The pre-clinical in vivo studies in rabbits demonstrated the supremacy of optimized LCNs in alleviating glaucoma following ocular application compared to the marketed product (Travatan^®^). Such a therapeutic modality represents a worthwhile option to boost the efficacy of anti-glaucoma drugs, awaiting further pre-clinical studies in other animals, such as monkey models, and clinical translation in human beings to validate the effectiveness of these tailored nanoparticles, which would offer a better therapeutic alternative than conventional ophthalmic delivery systems.

## Figures and Tables

**Table 1:** The results of dependent variables of the prepared liquid crystalline nanostructures formulae based on the D-optimal design.

Formula Code	Factor A	Factor B	Factor C	Responses * ± SD		
	Stabilizer Amount (mg)	PE Type	Stabilizer Type	Y1: PS (nm)	Y2: PDI	Y3: ZP (mV)
F1	1.25	Capmul^®^ MCM	TPGS	346.45 ± 30.05	0.40 ± 0.04	−40.80 ± 5.05
F2	1.25	None	Tween 80	666.35 ± 60.83	0.50 ± 0.05	−41.15 ± 2.34
F3	21.4375	Captex^®^ 8000	Tween 80	182.53 ± 24.43	0.43 ± 0.09	−36.58 ± 1.13
F4	25	Oleic acid	TPGS	232.55 ± 38.41	0.46 ± 0.01	−74.75 ± 7.34
F5	25	None	Tween 80	260.80 ± 57.46	0.42 ± 0.08	−31.50 ± 0.42
F6	11.9375	Captex^®^ 8000	TPGS	205.05 ± 9.57	0.38 ± 0.02	−36.98 ± 1.50
F7	4.8125	Captex^®^ 8000	P407	160.38 ± 5.05	0.15 ± 0.04	−20.58 ± 0.46
F8	25	None	TPGS	109.08 ± 6.53	0.36 ± 0.06	−26.20 ± 2.51
F9	20.25	Capmul^®^ MCM	P407	150.70 ± 7.51	0.32 ± 0.07	−17.85 ± 1.16
F10	1.25	Oleic acid	P407	160.63 ± 10.95	0.32 ± 0.04	−66.65 ± 5.72
F11	1.25	Captex^®^ 8000	Tween 80	561.25 ± 66.22	0.57 ± 0.05	−52.78 ± 9.94
F12	1.25	Oleic acid	Tween 80	176.03 ± 5.91	0.37 ± 016	−72.70 ± 6.88
F13	17.875	None	P407	307.35 ± 36.12	0.61 ± 0.07	−19.28 ± 1.84
F14	4.8125	Oleic acid	TPGS	226.80 ± 22.52	0.42 ± 0.04	−81.03 ± 7.71
F15	21.675	Oleic acid	P407	333.83 ± 51.39	0.47 ± 0.03	−37.18 ± 2.88
F16	4.21875	Capmul^®^ MCM	Tween 80	579.53 ± 229.65	0.58 ± 0.17	−41.53 ± 3.81
F17	25	Captex^®^ 8000	P407	147.78 ± 11.14	0.32 ± 0.05	−15.18 ± 0.95
F18	25	Capmul^®^ MCM	Tween 80	418.25 ± 117.58	0.39 ± 0.07	−26.40 ± 1.27
F19	8.375	None	TPGS	180.75 ± 29.95	0.33 ± 0.05	−29.48 ± 1.91
F20	21.4375	Capmul^®^ MCM	TPGS	195.28 ± 17.76	0.41 ± 0.04	−32.93 ± 1.54

* All data are mean of triplicates ± SD. SD—standard deviation; PS—particle size; PDI—polydispersity index; ZP—zeta potential; PE—penetration enhancer.

**Table 2:** ANOVA test results of all responses studied, according to the D-optimal design.

Terms	Responses					
	PS		PDI		ZP	
	*F*-Value	*p*-Value	*F*-Value	*p*-Value	*F*-Value	*p*-Value
Model	24.88 *	0.0393	30.8 *	0.0319	27.57 *	<0.0001
A	35.36 *	0.0271	0.0143 ^NS^	0.9156	11.32 *	0.0051
B	7.19 ^NS^	0.1245	10.81 ^NS^	0.0859	36.7 *	<0.0001
C	58.43 *	0.0168	51.71 *	0.019	16.79 *	0.0002
AB	15.64 ^NS^	0.0607	2.67 ^NS^	0.2846	-	-
AC	16.62 ^NS^	0.0567	46.22 *	0.0212	-	-
BC	16.1 ^NS^	0.0596	42.95 *	0.0229	-	-

A—stabilizer amount; B—PE type; C—stabilizer type; PS—particle size; PDI—polydispersity index; ZP—zeta potential. * Significant at 5% probability (*p* < 0.05). ^NS^ non-significant.

**Table 3:** Compositions, experimental and predicted data, and prediction error (%) of PS, PDI, and ZP responses of the optimized liquid crystalline nanostructures.

Formula Code	A: Stabilizer Amount (mg)	B: PE Type	C: Stabilizer Type	Experimental Results * ± SD			Predicted Results			Prediction Error (%)		
				PS (nm)	PDI	ZP (mV)	PS (nm)	PDI	ZP (mV)	PS	PDI	ZP
F-1-O	25	Oleic acid	Tween 80	238.11 ± 17.21	0.26 ± 0.01	−67.30 ± 2.81	207.32	0.22	−67.11	14.85	19.09	0.28
F-2-O	1.25	Oleic acid	P407	160.63 ± 8.80	0.32 ± 0.02	−66.65 ± 6.91	155.98	0.31	−60.07	2.98	1.29	10.96
F-3-O	25	Captex^®^ 8000	Tween 80	159.10 ± 10.98	0.39 ± 0.01	−32.40 ± 5.17	167.59	0.41	−34.02	5.07	3.67	4.75
F-4-O	25	Captex^®^ 8000	TPGS	143.05 ± 12.67	0.47 ± 0.04	−26.89 ± 2.10	119.54	0.40	−33.42	19.62	17.20	19.54
F-5-O	4.252	Captex^®^ 8000	P407	176.10 ± 9.74	0.17 ± 0.02	−21.30 ± 3.29	167.75	0.15	−25.44	4.98	18.49	16.27
F-6-O	1.25	Captex^®^ 8000	P407	205.50 ± 13.45	0.14 ± 0.03	−24.20 ± 2.12	171.77	0.12	−26.97	19.63	14.75	10.28
F-7-O	1.25	Capmul^®^ MCM	TPGS	346.45 ± 18.21	0.40 ± 0.02	−40.80 ± 1.17	336.90	0.41	−42.12	2.84	1.59	3.13
F-8-O	25	Capmul^®^ MCM	TPGS	167.03 ± 10.90	0.33 ± 0.04	−29.10 ± 3.22	187.60	0.40	−29.98	10.97	17.66	2.93

* All experimental data are mean of triplicates ± SD. SD—standard deviation; PS—particle size; PDI—polydispersity index; ZP—zeta potential; PE—penetration enhancer.

**Table 4:** The compositions and characterization results of TRAVO-loaded optimized LCNs.

Formula Code	A: Stabilizer Amount (mg)	B: PE Type	C: Stabilizer Type	Data * ± SD			
				PS (nm)	PDI	ZP (mV)	EE%
F-1-L	25	Oleic acid	Tween 80	216.20 ± 6.12	0.27 ± 0.03	−72.93 ± 1.97	85.30 ± 4.29
F-2-L	1.25	Oleic acid	P407	167.45 ± 8.54	0.33 ± 0.03	−62.65 ± 3.12	73.36 ± 15.54
F-3-L	25	Captex^®^ 8000	Tween 80	129.40 ± 11.73	0.34 ± 0.03	−17.55 ± 2.10	82.54 ± 7.65
F-4-L	25	Captex^®^ 8000	TPGS	245.85 ± 3.45	0.44 ± 0.05	−13.10 ± 1.27	71.29 ± 8.87
F-5-L	4.252	Captex^®^ 8000	P407	178.08 ± 11.59	0.18 ± 0.02	−19.45 ± 4.38	80.71 ± 3.68
F-6-L	1.25	Captex^®^ 8000	P407	231.35 ± 12.99	0.36 ± 0.01	−27.80 ± 1.27	75.16 ± 6.10
F-7-L	1.25	Capmul^®^ MCM	TPGS	361.57 ± 29.21	0.42 ± 0.04	−43.21 ± 7.22	84.31 ± 5.09
F-8-L	25	Capmul^®^ MCM	TPGS	212.85 ± 16.65	0.43 ± 0.02	−36.60 ± 3.45	77.20 ± 5.43

* All data are mean of triplicates ± SD. All formulae were loaded with Travoprost at a concentration of 40 µg/mL similar to the marketed product Travatan^®^. PS—particle size; PDI—polydispersity index; ZP—zeta potential; EE—entrapment efficiency; SD—standard deviation.

**Table 5:** The results of permeation parameters of TRAVO-loaded LCNs.

Formula Code	Data * ± SD	
	Jss (µg/cm^2^/h)	Kp (cm/h)
F-1-L	25.96 ± 2.05	0.64 ± 0.04
F-2-L	11.92 ± 3.07	0.29 ± 0.09
F-3-L	27.11 ± 3.25	0.67 ± 0.06
F-4-L	12.26 ± 1.54	0.30 ± 0.03
F-5-L	6.85 ± 0.57	0.17 ± 0.01
F-6-L	6.85 ± 0.57	0.14 ± 0.00
F-7-L	8.40 ± 1.05	0.21 ± 0.03
F-8-L	9.85 ± 0.97	0.24 ± 0.04
DS	1.53 ± 0.02	0.03 ± 0.00

* All data are mean of triplicates ± SD. Jss—steady-state flux; Kp—permeability coefficient; DS—drug solution.

**Table 6:** The percent reduction in intraocular pressure in the left rabbit’s eye was treated with the different prepared formulae F-1-L, F-3-L, and Travatan^®^ over 72 h.

Time (h)	Mean % IOP Reduction * ± SD		
	G-I: F-1-L	G-II: F-3-L	G-III: TRAVATAN^®^
0.5	0.00 ± 0.00	7.53 ± 0.00	14.52 ± 0.00
1	20.79 ± 6.18	14.52 ± 0.00	26.88 ± 0.00
2	37.90 ± 0.00	30.56 ± 6.36	37.90 ± 0.00
4	41.04 ± 5.43	42.65 ± 4.70	55.65 ± 0.00
6	58.06 ± 4.19	52.87 ± 4.81	59.41 ± 0.00
8	52.87 ± 4.81	51.52 ± 4.17	60.48 ± 4.19
10	52.87 ± 4.81	55.56 ± 3.90	59.41 ± 0.00
12	54.30 ± 2.33	58.06 ± 4.19	52.96 ± 2.33
24	51.52 ± 4.17	62.63 ± 6.05	39.52 ± 2.79
36	52.87 ± 4.81	54.30 ± 2.33	34.62 ± 2.88
48	39.34 ± 5.90	50.09 ± 4.81	29.11 ± 3.83
60	20.79 ± 6.18	48.57 ± 6.54	14.52 ± 0.00
72	2.51 ± 4.35	32.62 ± 2.44	0.00 ± 0.00

* All data are mean of triplicates ± SD. IOP—intraocular Pressure; SD—standard deviation. F-1-L is composed of 25 mg GMO, 25 mg oleic acid, and 25 mg Tween 80. F-3-L is composed of 25 mg GMO, 25 mg Captex^®^ 8000 and 25 mg Tween 80.

**Table 7:** The pharmacokinetic parameters of selected TRAVO-loaded LCNs formulae compared to Travatan^®^ measured in rabbits’ eye aqueous humor.

PK Parameters	Mean Data * ± SD		
	F-1-L	F-3-L	Travatan^®^
T_max_ (h)	2.00	6.00	1.00
C_max_ (ng/mL)	1.46 ± 0.06	1.80 ± 0.15	1.42 ± 0.09
AUC_0-48_ (ng.h/mL)	43.02 ± 2.97	62.77 ± 2.73	41.03 ± 1.63
AUC_inf_ (ng.h/mL)	133.63 ± 11.54	406.69 ± 17.12	125.98 ± 8.54
MRT (h)	11.73 ± 0.22	23.18 ± 0.57	11.58 ± 0.23
%F	106.10	322.82	-

* All data are mean of triplicates ± SD. Tmax: Time of the maximum concentration, Cmax: Maximum concentration, AUC: Area under the curve, MRT: Mean residence time, F = Relative bioavailability.

## Data Availability

The datasets generated during the current study are available from the corresponding authors upon request.

## Supplementary Materials

The following supporting information can be downloaded at: https://www.mdpi.com/article/10.3390/pharmaceutics15030954/s1, Table S1: Physical characterization of liquid crystalline nanostructure formulae prepared with different lipid types during the preliminary study. Table S2. Physical characterization of liquid crystalline nanostructure formulae prepared using different stabilizers during the preliminary study. Table S3. Physical characterization of liquid crystalline nanostructure formulae prepared using different stabilizer amounts during the preliminary study. Table S4. Physical stability data of the selected TRAVO-loaded LCNs stored under refrigeration at 5 ± 3 °C for 90 days. Table S5. Physical characteristics of the selected TRAVO-loaded LCNs after sterilization by gamma irradiation at different doses (References [62,63,64,65,66,67,68,69,70,71,72,73,74,75,76] are cited in the Supplementary Materials).

## Abbreviations

AUC—area under the curve; Cmax—maximum concentration; DLS—dynamic light scattering; DS—drug solution; EE%—entrapment efficiency; F%—relative bioavailability; FP receptor—prostaglandin F receptor; G—group; GMO—glyceryl monooleate; H&E—hematoxylin and Eosin; HPLC—high performance liquid chromatography; IOP—intraocular pressure; IS—internal standard; Jss—steady state flux; Kp—permeability coefficient; LCNs—liquid crystalline nanostructures; LDA—laser Doppler Anemometry; LOD—limit of detection; LOQ—limit of quantitation; MO—monoolein; MRM—multiple reactions monitoring; NPs—nanoparticles; NS—nano-sponge; PDI—polydispersity index; PE—penetration enhancer; PS—particle size; PYT—phytantriol; Q8—cumulative drug amount permeated after 8 h; SD—standard deviation; STF—simulated tear fluid; TBME—tertiary butyl-methyl ether; TEM—transmission electron microscopy; Tmax—time to reach maximum concentration; TPGS—tocopherol polyethylene glycol 1000 succinate; TRAVO—Travoprost; XRPD—X-ray powder diffraction; ZP—zeta potential.
