# Supplementary material for: Travoprost Liquid Nanocrystals: An Innovative Armamentarium for Effective Glaucoma Therapy
Source: Pharmaceutics. 2023 Mar 15;15(3):954. doi: 10.3390/pharmaceutics15030954 (PMC10056037; doi:10.3390/pharmaceutics15030954)
Supplement: Supplementary file 1 [file pharmaceutics-15-00954-s001.zip › pharmaceutics-2110788-supplementary.pdf]

pharmaceutics

Supplementary Travoprost Liquid Nanocrystals: An Innovative Armamentarium for Effective Glaucoma Therapy.

Mohamed A. El-Gendy; Mai Mansour; Mona I. A. El-Assal; Rania A. H. Ishak and Nahed D. Mortada

Preliminary studies

Preliminary studies were conducted preparing PE-based LCNs using Phytantriol (PYT) as the lipid compared to GMO, various types of stabilizers such as Cholesterol, Lecithin, TPGS, Solutol®HS, Brij52, MyrjS40 and Tween 80 in comparison to P407, and different stabilizer amounts. The PS, PDI and ZP were used as the characterization parameters in our preliminary trials to set the type and the amount range of the lipid and stabilizer to be used in the subsequent experimental design. The results are presented in **Tables S1-S3**

1. Effect of lipid type

First, the effect of lipid type was studied by preparing different LCNs using either GMO or PYT, both in the presence of P407 as stabilizer and one of the different PE types selected either oleic acid, Captex® 8000 or Capmul® MCM**.** The amounts of formulation components, either lipid, stabilizer, or PE, were maintained constant at 25 mg each. As shown in **Table S1**, LCNs prepared with GMO showed significant lower PS than those with PYT (p<0.05) for the same PE type as the results ranged between 139.91 ± 5.11 to 259.53 ± 19.51 nm and 165.37 ± 8.68 to 424.43 ± 62.27 nm, respectively. Our data were in agreement with previous reports, as GMO systems produced significant lower PS than those prepared using PYT [62 – 64]. This could be attributed to the high melting point of PYT (56-57°C) compared to that of GMO (35°C), which leads to higher viscosity of the dispersion medium and hence decreasing efficiency of homogenization process in reducing PS of the formed nanoparticles [65].

Similarly, LCNs prepared with GMO showed homogenous size distribution relative to PYT-LCNs for the same type of PE where the respective PDI values were in the range of 0.10 ± 0.09 to 0.31 ± 0.02, and 0.23 ± 0.04 to 0.58 ± 0.09, as presented in **Table S1**. In contrast, the surface charges of the prepared LCNs were comparable irrespective to the lipid type revealing a non-significant difference between the obtained data for the same PE type (p>0.05). The obtained results deduced that the type of PE is the influential factor for the difference in surface charges. As shown in **Table S1**, Oleic acid provided higher negative ZP values to the formed LCNs than Captex® 8000 and Capmul® MCM did. The negative charges of the free fatty acid are mostly responsible for the increase in ZP [39]. Because the latter two mono-/di-glycerides are amphiphilic, they are most probably adsorbed onto the surfaces of the formed NPs, resulting in a shielding effect with a considerable decrease in ZP magnitudes [49]. According to the previous findings, PYT was excluded, and GMO was maintained as the lipid of choice during the subsequent experiments confirming its suitability in the preparation of PE-enriched LCNs.

Table S1. Physical characterization of liquid crystalline nanostructure formulae prepared with different lipid types during the preliminary study.

Formula Code	Lipid Type	PE Type	PS (nm)* ± SD	PDI* ± SD	ZP (mV)* ± SD
RF-1	GMO	Oleic acid	259.53 ± 19.51	0.31 ± 0.02	-24.90 ± 0.56
TF-1	PYT		424.43 ± 62.27	0.58 ± 0.09	-24.26 ± 0.40
RF-2	GMO	Captex® 8000	170.43 ± 6.21	0.12 ± 0.00	-8.08 ± 0.09
TF-2	PYT		243.40 ± 9.61	0.23 ± 0.04	-10.50 ± 4.33
RF-3	GMO	Capmul® MCM	139.91 ± 5.11	0.10 ± 0.09	-9.66 ± 0.24
TF-3	PYT		165.37 ± 8.68	0.43 ± 0.13	-8.19 ± 1.22

All data are mean of triplicates ± SD.
RF: Reference Formula, TF: Trial Formula, PE: Penetration Enhancer, SD: Standard Deviation, PS: Particle Size, PDI: Polydispersity Index, ZP: Zeta Potential
All formulae composed of 25 mg of each lipid type (GMO or PYT), 25 mg P407 and 25 mg PE (Oleic acid, Captex 8000® or Capmul MCM®)

2. Effect of stabilizer type

GMO-based nanostructures were screened using different stabilizers *viz*; Cholesterol, Lecithin, TPGS, Solutol®HS, Brij52, MyrjS40 and Tween 80, relative to P407. For better comparison, LCN formulations were all prepared at the same GMO: stabilizer mass ratio (1:1) and without the inclusion of any PE. The results of characterization parameters of these formulae are summarized in Table S2.

Regarding the PS results shown in **Table S2**, the prepared LCNs showed variable sizes ranging from 106.3 ± 0.98 to 7730 ± 0.76 nm. The LCNs could be ascendingly arranged in terms of PS according to the type of stabilizer used as follows; TPGS < Tween < Lecithin < P407 < Cholesterol < Myrj < Solutol < Brij. TPGS and Tween 80 exhibited the smallest PS among other stabilizers; as the respective data are 106.3 ± 0.98 and 179.6 ± 4.87 nm, respectively, while Brij52 showed the largest ones with a PS of 7730 ± 0.76 nm. The PDI data were also found variable ranging from 0.29 ± 0.02 to 1 ± 0.00. TPGS and Tween80 also manifested the lowest PDI values of 0.29 ± 0.02 and 0.330 ± 0.02, respectively confirming a uniform LCN size distribution. All the investigated stabilizers are characterized by high HLB values (greater than 8), which favors the formation of o/w emulsions [66],[67], except for Lecithin and cholesterol which possess low HLB values of less than 8. Thus, it can be inferred that HLB value might not be the key influential parameter for PS variation between the different stabilizers, so the correlation with the chemical structure of each type could be of an interest.

Table S2. Physical characterization of liquid crystalline nanostructure formulae prepared using different stabilizers during the preliminary study.

Formula Code	Stabilizer Type	PS* (nm) ± SD	PDI* ± SD	ZP* (mV) ± SD
**RF-4**	**P407**	224.40 ± 13.70	0.39 ± 0.08	-13.80 ± 0.91
**TF-4**	**Cholesterol**	335.00 ± 3.04	0.40 ± 0.00	-34.70 ± 0.63
**TF-5**	**Lecithin**	203.10 ± 3.11	0.43 ± 0.00	-59.30 ± 4.67
**TF-6**	**TPGS**	106.30 ± 0.98	0.29 ± 0.02	-14.00 ± 1.56
**TF-7**	**Solutol®**	603.00 ± 7.63	0.66 ± 0.05	-31.50 ± 0.07
**TF-8**	**Brij58**	7730.00 ± 0.76	1.00 ± 0.00	-35.20 ± 2.05
**TF-9**	**MyrjS40**	461.60 ± 65.90	0.50 ± 0.02	-35.30 ± 1.20
**TF-10**	**Tween 80**	179.60 ± 4.87	0.33 ± 0.02	-19.60 ± 0.63

* All data are mean of triplicates ± SD.
RF: Reference Formula, TF: Trial Formula, SD: Standard Deviation, PS: Particle Size, PDI: Polydispersity Index, ZP: Zeta Potential
All formulae were prepared with GMO and stabilizer maintained at a mass ratio (1:1) with 25 mg each and without the inclusion of PE.

As noticed, the steroid-based lipid compound 'cholesterol' revealed a rigid bulky structure that might explain the prominent increase in size of its corresponding LCNs. However, the other tested surfactants demonstrated analogous linear structures composed of long alky chains convenient for good emulsification and steric repulsion. Hence, the discrepancy in the size results would emphasize the importance of selecting the optimal type and concentration of stabilizers considering sufficient affinity for the LCN surfaces and adequate interface coverage which depends on particle/surfactant ratio [68]. The fact might also be elucidated to the changes in surfactant packing that occur at the o/w interface upon lipid crystallization after homogenization and cooling processes during manufacture [69].

It can be noticed from **Table S2** that TPGS showed the lowest ZP value of -14 ± 1.56 mV, while lecithin showed the highest one reaching -59.3 ± 4.67 mV. Kulkarni and Feng, 2013 studied the effect of TPGS coating on the prepared nanoparticles (NPs), then measured the PS, PDI and ZP before and after TPGS coating [70]. They found that ZP values was shifted towards more positive direction after surface coating, suggesting that the neutral charge of the adsorbed surface layer which was probably masking the particle surface charge and force shear plane further away from NP surfaces, hence reducing the ZP relative to Stern potential. On the other hand, lecithin is an anionic surfactant with an electronegative charge, which led to the increase in ZP values [71]. Most of the formulae showed absolute ZP values higher than 10 mV. According to literature, ZP values higher than ± 10 mV yields a relatively stable colloidal system due to the electric repulsion among particles which is a key property of an agglomeration resistant dispersion [72] Based

on the previous results, Tween 80 and TPGS were chosen along with P407 as the optimum stabilizers for the preparation of LCNs as they showed the lowest PS with acceptable PDI and ZP values.

3. Effect of stabilizer amount

After stabilizer selection, our goal was to determine its minimum amount that can be needed for the successful formation of stable LCNs, since this is of great importance in pharmaceutical industries. Initially, P407 was investigated to stabilize LCNs at varying tiny amounts less than 25 mg, with and without the inclusion of PE, aiming to set the lowest amount required to stabilize liquid crystals. **Table S3** gathers the characterization data of these LCN formulations.

Table S3. Physical characterization of liquid crystalline nanostructure formulae prepared using different stabilizer amounts during the preliminary study.

Formula	PE Type	P407 amount (mg)	PS* (nm) ± SD	PDI* ± SD	ZP* (mV) ± SD
RF-4	None	25	224.40 ± 13.70	0.39 ± 0.08	-13.80 ± 0.91
TF-11		5	267.00 ± 21.71	0.40 ± 0.04	-16.80 ± 0.07
TF-12		2.5	288.00 ± 16.90	0.43 ± 0.11	-18.00 ± 0.21
TF-13		1.25	451.40 ± 52.67	0.50 ± 0.06	-24.60 ± 0.07
TF-14		0	NA	NA	NA
RF-1	Oleic acid	25	259.53 ± 19.51	0.31 ± 0.02	-24.90 ± 0.56
TF-15		5	389.00 ± 77.78	0.50 ± 0.15	-46.20 ± 5.23
TF-16		1.25	160.63 ± 10.95	0.32 ± 0.04	-66.65 ± 5.72
TF-17		0	NA	NA	NA
RF-2	Captex® 8000	25	170.43 ± 6.21	0.12 ± 0.00	-8.08 ± 0.09
TF-18		5	187.00 ± 4.38	0.19 ± 0.01	-12.90 ± 0.07
TF-19		1.25	276.00 ± 6.78	0.40 ± 0.01	-14.60 ± 0.21
TF-20		0	NA	NA	NA
RF-3	Capmul® MCM	25	139.91 ± 5.11	0.10 ± 0.09	-9.66 ± 0.24
TF-21		5	189.50 ± 0.70	0.30 ± 0.00	-17.30 ± 0.35
TF-22		1.25	267.90 ± 2.97	0.31 ± 0.00	-15.60 ± 0.21
TF-23		0	NA	NA	NA

*All data are mean of triplicates ± SD.
RF: Reference Formula, TF: Trial Formula, PE: Penetration Enhancer, SD: Standard Deviation, PS: Particle Size, PDI: Polydispersity Index, ZP: Zeta Potential, NA: Not applicable. All formulae were composed of 25 mg of GMO and 25 mg PE (if present) at different P407 amounts.

Firstly, it can be noticed from **Table S3**, that the absence of stabilizer resulted in the complete failure to form LCNs. Despite the fact that LCNs are thermodynamically stable, when dispersed in aqueous media, the liquid crystals tend to aggregate as a result of hydrophobic regions being exposed to the external hydrophilic aqueous medium. As a result, incorporation of stabilizing agents becomes critical step in LCNs preparation to prevent dispersed particles from recoalescing when dispersed in water. The stabilizer's primary role is to create an electrostatic and steric barrier between particles, preventing close particle interaction and preserving the dispersed particles in a stable state. This action is achieved by the stabilizer participating in the lipid water assembly without disrupting the cubic liquid crystallinity, therefore selecting an optimum amount of stabilizer is crucial [73].

By inspection of **Table S3**, it can be observed that the increase in P407 amounts from 1.25 to 25 mg led to the formation of PE-free LCNs with significantly ($P<0.05$) reduced PS; 451.40 ± 52.67 and 224.40 ± 13.70, respectively, noticing the non-significant difference in PS when raising the stabilizer amount from 2.5 to 25 mg ($p>0.05$). Moreover, the least amount of P407 (1.25 mg) has been shown to be sufficient to stabilize all PE-containing LCNs, where the PS results attained 160.63 ± 10.95, 276.00 ± 6.78, and 267.90 ± 2.97 nm in case of oleic acid, Captex® 8000 and Capmul® MCM, respectively. This could be due to the emulsifying properties of the PEs used [74],[75]. Also, it is worthy to notice the negligeable differences between the measured LCN sizes while using 5 and 25 mg of P407 in different formulae, this endorses the prominent stabilization effect of P407 even at lower amounts.

The obtained PDI values confirmed the size homogeneity of all the produced LCNs as they ranged from 0.1 to 0.5. As previously elucidated, the ZP data varied according to the type of PE used; where oleic acid-based particles showed the highest ZP range compared to those containing Captex® 8000 and Capmul® MCM. The previous findings could be due to the fact that high surfactant concentration decreases surface tension and thus stabilizes newly developed surfaces during homogenization and so leads to the production of smaller particles [76]. Likewise, the insufficient amount of surfactant may cause instability and recrystallization of lipid nanostructures [69]. Based on the obtained results, a minimum-maximum range of stabilizer amount was set at 1.25 mg – 25 mg during the subsequent experimental design.

Table S4. Physical stability data of the selected TRAVO-loaded LCNs stored under refrigeration at 5 ± 3°C for 90 days.

Formula Parameters* ± SD	F-1-L			F-3-L		
	Day 0	Day 30	Day 90	Day 0	Day 30	Day 90
PS (nm)	216.20 ± 6.12	212.00 ± 3.62	243.30 ± 2.62	129.40 ± 11.73	136.06 ± 7.09	144.40 ± 1.78
PDI	0.27 ± 0.03	0.31 ± 0.03	0.33 ± 0.01	0.34 ± 0.03	0.34 ± 0.04	0.34 ± 0.03
ZP (mV)	-72.93 ± 1.97	-69.31 ± 2.64	-68.70 ± 1.91	-17.55 ± 2.10	-15.15 ± 0.31	16.85 ± 0.21
EE (%)	85.30 ± 4.29	80.30 ± 4.29	81.03 ± 3.90	82.54 ± 7.65	83.54 ± 7.65	79.07 ± 2.65

* All data are mean of triplicates ± SD.
PS: Particle size, PDI: Polydispersity index, ZP: Zeta potential, EE: Entrapment efficiency, SD: Standard deviation.
F-1-L is composed of 25 mg GMO, 25 mg Oleic acid, and 25 mg Tween 80.
F-3-L is composed of 25 mg GMO, 25 mg Captex® 8000, and 25 mg Tween 80

Table S5. Physical characteristics of the selected TRAVO-loaded LCNs after sterilization by gamma irradiation at different doses.

Formula Parameters)* ± SD	F-1-L					F-3-L				
	Non-sterilized	5 KGy	10 KGy	15 KGy	25 KGy	Non-sterilized	5 KGy	10 KGy	15 KGy	25 KGy
PS (nm)	216.20 ± 6.12	218.31 ± 4.02	215.01 ± 2.82	221.30 ± 4.13	219.09 ± 7.34	129.40 ± 11.73	135.80 ± 7.64	125.87 ± 5.86	128.87± 3.73	134.98 ± 8.32
PDI	0.27 ± 0.03	0.26 ± 0.07	0.28 ± 0.01	0.31 ± 0.08	0.27 ± 0.10	0.34 ± 0.03	0.31 ± 0.04	0.33 ± 0.03	0.30 ± 0.08	0.36 ± 0.06
ZP (mV)	-72.93 ± 1.97	-74.03 ± 3.17	-70.93 ± 2.65	-72.04 ± 2.01	-70.43 ± 4.07	-17.55 ± 2.10	-15.53 ± 3.91	-19.21 ± 4.32	-20.66 ± 4.87	-18.42 ± 2.22
EE (%)	85.30 ± 4.29	83.00 ± 5.87	87.30 ± 6.09	82.06 ± 7.53	84.89 ± 6.03	82.54 ± 7.65	84.32 ± 8.73	79.21 ± 6.60	86.94 ± 7.11	80.04 ± 5.42

* All data are mean of triplicates ± SD.
PS: Particle size, PDI: Polydispersity index, ZP: Zeta potential, EE: Entrapment efficiency, SD: Standard deviation.
F-1-L is composed of 25 mg GMO, 25 mg Oleic acid, and 25 mg Tween 80.
F-3-L is composed of 25 mg GMO, 25 mg Captex® 8000, and 25 mg Tween 80.

References

39. Mansour, M.; Kamel, A.O.; Mansour, S.; Mortada, N.D. Novel Polyglycerol-Dioleate Based Cubosomal Dispersion with Tailored Physical Characteristics for Controlled Delivery of Ondansetron. *Colloids Surf. B Biointerfaces* **2017**, *156*, 44–54.
49. Wu, S.; Wang, G.; Lu, Z.; Li, Y.; Zhou, X.; Chen, L.; Cao, J.; Zhang, L. Effects of Glycerol Monostearate and Tween 80 on the Physical Properties and Stability of Recombined Low-Fat Dairy Cream. *Dairy Sci. Technol.* **2016**, *96*, 377–390.
62. Peng, X.; Zhou, Y.; Han, K.; Qin, L.; Dian, L.; Li, G.; Pan, X.; Wu, C. Characterization of Cubosomes as a Targeted and Sustained Transdermal Delivery System for Capsaicin. *Drug Des. Devel. Ther.* **2015**, *9*, 4209–4218. https://doi.org/10.2147/DDDT.S86370.
63. Shi, X.; Peng, T.; Huang, Y.; Mei, L.; Gu, Y.; Huang, J.; Han, K.; Li, G.; Hu, C.; Pan, X.; et al. Comparative Studies on Glycerol Monooleate- and Phytantriol-Based Cubosomes Containing Oridonin in Vitro and in Vivo. *Pharm. Dev. Technol.* **2017**, *22*, 322–329. https://doi.org/10.3109/10837450.2015.1121496.
64. Hong, L.; Dong, Y.-D.; Boyd, B.J. Preparation of Nanostructured Lipid Drug Delivery Particles Using Microfluidic Mixing. *Pharm. Nanotechnol.* **2019**, *7*, 484–495. https://doi.org/10.2174/2211738507666191004123545.

65. Elmowafy, M.; Samy, A.; Raslan, M.A.; Salama, A.; Said, R.A.; Abdelaziz, A.E.; El-Eraky, W.; el Awdan, S.; Viitala, T. Enhancement of Bioavailability and Pharmacodynamic Effects of Thymoquinone Via Nanostructured Lipid Carrier (NLC) Formulation. *AAPS PharmSciTech* **2016**, *17*, 663–672. https://doi.org/10.1208/S12249-015-0391-0.
66. Kassem, M.G.A.; Ahmed, A.M.M.; Abdel-Rahman, H.H.; Moustafa, A.H.E. Use of Span 80 and Tween 80 for Blending Gasoline and Alcohol in Spark Ignition Engines. *Energy Rep.* **2019**, *5*, 221–230. https://doi.org/10.1016/J.EGYR.2019.01.009.
67. Kumar, G.P.; Rajeshwarrao, P. Nonionic Surfactant Vesicular Systems for Effective Drug Delivery—An Overview. *Acta Pharm. Sin. B* **2011**, *1*, 208–219. https://doi.org/10.1016/J.APSB.2011.09.002.
68. Young, T.J.; Johnston, K.P.; Pace, G.W.; Mishra, A.K. Phospholipid-Stabilized Nanoparticles of Cyclosporine a by Rapid Expansion from Supercritical to Aqueous Solution. *AAPS PharmSciTech* **2004**, *5*, 70–85. https://doi.org/10.1208/PT050111.
69. Helgason, T.; Awad, T.S.; Kristbergsson, K.; McClements, D.J.; Weiss, J. Effect of Surfactant Surface Coverage on Formation of Solid Lipid Nanoparticles (SLN). *J. Colloid Interface Sci.* **2009**, *334*, 75–81. https://doi.org/10.1016/J.JCIS.2009.03.012.
70. Kulkarni, S.A.; Feng, S.S. Effects of Particle Size and Surface Modification on Cellular Uptake and Biodistribution of Polymeric Nanoparticles for Drug Delivery. *Pharm. Res.* **2013**, *30*, 2512–2522. https://doi.org/10.1007/S11095-012-0958-3.
71. Yegin, Y.; Oh, J.K.; Akbulut, M.; Taylor, T. Cetylpyridinium Chloride Produces Increased Zeta-Potential on Salmonella Typhimurium Cells, a Mechanism of the Pathogen's Inactivation. *npj Sci. Food* **2019**, *3*, 1–7. https://doi.org/10.1038/s41538-019-0052-x.
72. Tlijani, M.; Lassoued, M.A.; Bahloul, B.; Sfar, S. Development of a BCS Class II Drug Microemulsion for Oral Delivery: Design, Optimization, and Evaluation. *J. Nanomater.* **2021**, *2021*. https://doi.org/10.1155/2021/5538940.
73. Spicer, P.T.; Hayden, K.L.; Lynch, M.L.; Ofori-Boateng, A.; Burns, J.L. Novel Process for Producing Cubic Liquid Crystalline Nanoparticles (Cubosomes). *Langmuir* **2001**, *17*, 5748–5756. https://doi.org/10.1021/la010161w.
74. Choulis, N.H. Miscellaneous Drugs, Materials, Medical Devices, and Techniques. *Side Eff. Drugs Annu.* **2011**, *33*, 1009–1029. https://doi.org/10.1016/B978-0-444-53741-6.00049-0.
75. Prajapati, H.N.; Dalrymple, D.M.; Serajuddin, A.T.M. A Comparative Evaluation of Mono-, Di- and Triglyceride of Medium Chain Fatty Acids by Lipid/Surfactant/Water Phase Diagram, Solubility Determination and Dispersion Testing for Application in Pharmaceutical Dosage Form Development. *Pharm. Res.* **2012**, *29*, 285–305. https://doi.org/10.1007/S11095-011-0541-3.
76. McClements, D.J. Crystals and Crystallization in Oil-in-Water Emulsions: Implications for Emulsion-Based Delivery Systems. *Adv. Colloid Interface Sci.* **2012**, *174*, 1–30. https://doi.org/10.1016/J.CIS.2012.03.002.
